# Dynamic functional assessment of T cells reveals an early suppression correlating with adverse outcome in polytraumatized patients

**DOI:** 10.3389/fimmu.2025.1538516

**Published:** 2025-03-24

**Authors:** Tobias Jooss, Katharina Maier, Lena-Marie Reichardt, Bianca Hindelang, Lönna Süberkrüb, Kim Lena Hamberger, Jasmin Maria Bülow, Konrad Schuetze, Florian Gebhard, Marco Mannes, Rebecca Halbgebauer, Lisa Wohlgemuth, Markus Huber-Lang, Borna Relja, Christian B. Bergmann

**Affiliations:** ^1^ Translational and Experimental Trauma Research, Department of Trauma-, Hand-, Plastic- and Reconstructive Surgery, Ulm University Medical Center, Ulm, Germany; ^2^ Department of Trauma-, Hand-, Plastic- and Reconstructive Surgery, Ulm University Medical Center, Ulm, Germany; ^3^ Institute of Clinical and Experimental Trauma Immunology, Ulm University Medical Center, Ulm, Germany

**Keywords:** trauma, immunomonitoring, T cells, interferon gamma, CD8 T cells, prediction

## Abstract

**Introduction:**

Most trauma patients require intensive care treatment and are susceptible to developing persistent inflammation and immunosuppression, potentially leading to multi organ dysfunction syndrome (MODS) and dependence on long term care facilities. T cells undergo changes in numbers and function post trauma. T cell dysfunction in polytraumatized patients was characterized using functional immunomonitoring to predict individual clinical outcome. Moreover, the potential to reverse T cell dysfunction using Interleukin (IL)-7 was examined.

**Methods:**

Blood samples were drawn from healthy individuals and prospectively enrolled polytrauma patients (Injury Severity Score ≥ 18) on admission, 8, 24 and 48 hours, 5 and 10 days after. CD3/28-stimulated cytokine production of T cells in whole blood was assessed via Enzyme Linked Immuno Spot (ELISpot). T cell subsets were quantified via counting and flow cytometry. Unfavorable physical performative outcome was defined as death or new functional disability necessitating long term care. Secondary outcomes were the development of MODS and in-hospital mortality. IL-7 was added ex vivo to test reversibility of cytokine disturbances.

**Results:**

34 patients were enrolled. The different outcome groups showed no difference in injury severity. Patients with favorable physical performative outcome revealed higher functional T cell specific Interferon γ (IFN-γ) and IL-17 (8 hours) and lower IL-10 production (day 5) and higher CD8 T cell concentrations. Patients without MODS development showed a higher IFN-γ (day 10), higher IL-2 (8 hours) and higher IL-17 production (admission, day 5). There were no differences regarding in-hospital mortality. Systemic blood IFN-γ, IL-2 and IL-10 concentrations only correlated with MODS (24 hours). Systemic CD8 T cell numbers correlated with functional IFN-γ production. Whole blood stimulation with IL-7 increased functional T cell IFN-γ release.

**Discussion:**

Our study reveals an early characteristic overall T cell dysfunction of pro-inflammatory (IFN-γ, IL-2, IL-17) and immunosuppressive (IL-10) subtypes in polytraumatized patients. Our data indicates that rather the functional capacity of T cells to release cytokines, but not systemic cytokine concentrations can be used to predict outcome post trauma. We assume that the early stimulation of pro- and anti-inflammatory T cells benefits polytraumatized patients. Potentiation of functional IFN-γ release might be achieved by IL-7 administration.

## Graphical Abstract

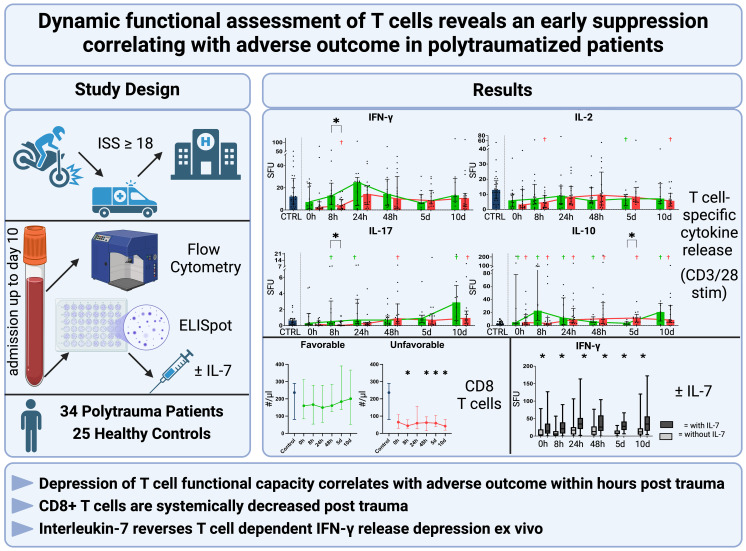

## 1 Introduction

According to the global burden of disease survey, traumatic injuries are responsible for over 200 million disability-adjusted life years lost worldwide and trauma accounts for 8% of all deaths in the world (1). Polytrauma is defined as a simultaneous injury to several body regions or organ systems, of which at least one or more injuries in combination are life-threatening (2). Up to 55% of polytraumatized patients die before reaching the hospital (3) and up to 84% of those that initially survive require intensive care treatment (4) of which a significant number will develop chronic critical illness, with prolonged stay in the intensive care unit (ICU), persistent organ dysfunction and persistent physical impairment (5, 6). Many traumatized patients under intensive care develop an immunological dysfunction leading to multi organ dysfunction syndrome (MODS) (7) and persistent inflammation and immunosuppression syndrome (PICS) (8). PICS is associated with high mortality and morbidity and causes these patients to become dependent on long term care facilities (8, 9).

In trauma patients, development of MODS along with other adverse outcomes is associated with functional and numerical depression of circulating lymphocytes in the patients’ blood, with CD8 T cells and NKT cells being numerically decreased in particular (10–12). The exact mechanisms that lead to this decrease are not fully understood. However, it has been shown that T cells become apoptotic and anergic post-trauma (13). Both lymphopenia and T cell dysfunction increase the risk for infections, which could contribute to the risk of MODS and mortality (14).

The investigation of T-helper (Th) cell specific cytokine release in trauma patients revealed conflicting results: Patients presenting with hemorrhagic shock showed a trend toward higher cytokine concentrations for the Th2-specific cytokines IL-4 and IL-10 with lower concentrations for the Th1-specific cytokines Interferon γ (IFN-γ) and IL-2 (15). Contrary to this, others could not find differences in serum Th1 or Th2 cytokine levels in trauma patients (16), highlighting the need to further characterize the T cell response after trauma.

Several studies investigated the dynamic of the immune response to trauma by measuring systemic cytokine concentrations (17–19). A portion of these studies have shown that certain cytokines, particularly IL-6, IL-8, and IL-10, correlate with mortality and organ dysfunction in critically ill patients (20, 21). These cytokines, along with others like inducible protein 10 and macrophage inflammatory protein-1β, may serve as early predictors of multiple organ failure in trauma patients (21). However, the clinical utility of most cytokine measurements remains limited due to lack of sensitivity and specificity, with only IL-6 and procalcitonin approaching routine clinical use (22, 23). Therefore it has been proposed that monitoring of immune cell function, rather than systemic cytokine concentrations, may better predict adverse outcome in trauma (24). We and others could recently show that functional immunomonitoring can predict mortality in a human sepsis population when measuring stimulated IFN-γ release using an Enzyme-Linked ImmunoSpot (ELISpot) Assay (25, 26). What has not been shown is whether that applies to a trauma population as well and if the release of other Th-specific cytokines is functionally impaired in trauma. Next, we do not know what pattern of functional T cell cytokine release is associated with either physical performative outcome, the development of MODS or survival in trauma patients. Therefore, this study aims to examine the functional T cell response to trauma specifically.

To combat immune dysregulation after trauma, various immune modulatory therapies have been proposed (27) but overall no therapeutic option has entered widespread clinical use. IL-7 plays a crucial role in T cell lymphopoiesis and survival (28), is being tested in several sepsis trials to combat lymphopenia (ClinicalTrials.gov ID: NCT02640807) and has shown beneficial results in a case report of a trauma patient suffering from intractable fungal wound sepsis (29). We therefore aim to test ex vivo if IL-7 alters the functional cytokine response in T cells in a beneficial manner.

This study seeks to apply functional immunomonitoring using ELISpot to examine functional changes in T cell specific cytokine release in polytraumatized patients to characterize T cell dynamics. We hypothesize that characterizing the functional capacity of T cells to release cytokines, namely IFN-γ, IL-2, IL-10 and IL-17 (30–32), predicts beneficial or adverse discharge conditions, the development of MODS and in-hospital mortality and therewith provide a prognostic method in polytrauma patients. Additionally, we aim to reveal systemic T cell numbers to assess whether functional cytokine release can be attributed to CD4 or CD8 T cells. Lastly, we will examine how IL-7 administration ex vivo alters cytokine release.

## 2 Methods

### 2.1 Clinical study

Polytraumatized patients were enrolled between May 2022 and May 2024 in a prospective observational study at the academic level 1 trauma center of the University Hospital Ulm, Germany. Patients were enrolled on admission and were followed up until hospital discharge. Ethics committee approval was granted by the respective regulatory body (No. 65/20 and 260/22 Ethics Committee of Ulm University).

#### 2.1.1 Patient screening

Blood was drawn from 34 polytraumatized patients ≥ 18 years old with an estimated injury severity score (ISS) ≥ 18 and immediately processed. The ISS was assessed after the initial CT scan on admission. Exclusion criteria for this study were: known infectious diseases like HIV or Hepatitis, radiation or chemotherapy within the 3 months leading up to admission, immune-suppressive medication, as well as prehospital cardiac arrest and pregnancy. Patients who were suspected not to survive the first 24 hours after admission were not enrolled. Patients who later had a confirmed ISS under 18 were subsequently excluded from analysis. Patient consent was obtained in written form from the patient or a legal guardian.

Twenty-five healthy control subjects who volunteered to have their blood drawn and analyzed in the same manner as the polytrauma patients were included. Healthy controls were matched to the patient cohort regarding age and sex.

#### 2.1.2 Blood sampling

Blood samples were obtained upon admission in the emergency department (0 hour timepoint), then 8, 24 and 48 hours, as well as 5 and 10 days after, while allowing for a 10% time variance at each timepoint. Blood was obtained either by peripheral venipuncture or via a central venous or arterial catheter and stored in different tubes (Ethylenediaminetetraacetic acid (EDTA) (flow cytometry, automated differential blood count), trisodium citrate (ELISpot assay) or silica granulate (serum cytokine analysis) respectively (all three tubes: Sarstedt AG, Nuembrecht, Germany)). The tubes were kept on ice during transport to the laboratory and the blood was then immediately used to conduct an ELISpot assay to assess immune function as well as flow cytometry for phenotyping. At each timepoint, an automated differential blood count using the EDTA blood was performed by the hospital Central Facility for Clinical Chemistry. In order to measure serum concentrations of various cytokines, blood samples in the silica granulate tubes were centrifuged for 15 minutes at 2000 g at 4°Celsius (C) and serum aliquots were immediately stored at -80°C until analysis.

### 2.2 Outcomes

The primary endpoint for this study was physical performative outcome of the patients at the time of discharge. Unfavorable physical performative outcome was defined either as in-hospital mortality or new admission into a short- or long-term skilled nursing facility, in-patient rehab facility or to another hospital. Favorable physical performative outcome was defined as discharge to the patient’s home with or without services. Secondary endpoints were the development of MODS, defined as a Sequential Organ Failure Assessment (SOFA) Score of 6 or more, on at least 2 consecutive days, more than 48 hours post admission (33) and in-hospital mortality.

### 2.3 T cell phenotyping

20 μl EDTA whole blood was stained for 30 minutes at 4°C in the dark, using the following mouse anti-human monoclonal antibodies with the respective fluorochromes: CD3-APC-H-7 (Clone SK7, BD Pharmingen, Eysins, Switzerland), CD4-FITC (Clone OKT4, BioLegend, San Diego, California, USA), CD8-PE-Cy7 (Clone SK1, BioLegend, San Diego, California, USA), CD45RA-PE (Clone HI100, eBioscience, Waltham, Massachusetts, USA), CCR7-APC (Clone G043H7, BioLegend, San Diego, California, USA) and CD56-PerCP-Cy5.5 (Clone MEM-188, BioLegend, San Diego, California, USA). After incubation, the cells were centrifuged at 340 g at 4°C for 5 minutes, washed using PBS, centrifuged again at 340 g at 4°C for 5 minutes and then FACS lysing solution (BD Biosciences, Eysins, Switzerland) was added according to the manufacturer’s instructions in order to lyse the red blood cells. After two more rounds of centrifugation at 340 g at 4°C for 5 minutes and washing with PBS, 100 μl of PBS containing 1% bovine serum albumin were added. The sample was then immediately acquired on a BD FACS Canto II flow cytometer (BD Biosciences, Eysins, Switzerland) and data was exported using Microsoft Excel, version 16.61.1 (Microsoft, Redmond, Washington, USA). T cells were defined as being CD3 positive and CD56 negative, and then separated into CD4 helper T cells and CD8 cytotoxic T cells. These subgroups were then differentiated based upon their expression of CCR7 and CD45Ra.

### 2.4 Serum cytokine analysis

Serum concentrations of IL-2, IL-17A, IL-10 as well as IFN-γ were measured using the LEGENDplex™ Human Essential Immune Response Panel (BioLegend, San Diego, California, USA, cat. number 740930) according to the instructions provided by the manufacturer.

### 2.5 ELISpot assay

In order to quantify cytokine production of T cells, 5 μl of the citrated whole blood per well were incubated at 37°C and 5% CO_2_ in a total of 200μl CTL-Test™ Medium (CTL, Shaker Heights, Ohio, USA) for 24 hours with a tolerance of +/- 4 hours, on polyvinylidene difluoride-backed ELISpot strips, precoated with either anti-IFN-γ, anti-IL-2, anti-IL-17 or anti-IL-10 capture antibodies (CTL, Shaker Heights, Ohio, USA). Before incubation, we added either a mix of anti-CD3 (0.5 μg/ml, Clone HIT3a, BioLegend, San Diego, California, USA) and anti-CD28 (5 μg/ml, Clone CD28.2, BioLegend, San Diego, California, USA) antibodies or anti-CD3/28 antibodies in combination with recombinant human IL-7 (50ng/ml, R&D Systems, Mineapolis, Minnesota, USA) to the blood in the incubation medium, while also leaving a sample unstimulated. After incubation, the plates were subsequently washed with room-temperature PBS and PBS with 0,05% Tween, and biotinylated secondary detection antibodies for the respective plate-bound cytokines (IFN-γ, IL-2, IL-17 or IL-10) were added. This mixture was left to incubate at room temperature for 2 hours on a plate shaker, after which the plates were washed again with PBS and Tween before adding streptavidin-bound alkaline phosphatase, which were left to incubate for 30 minutes at room temperature. Finally, developer solution was added as per the manufacturer’s instructions, before image capture and analysis. The prepared strips were scanned, analyzed and quality controlled for Spot Forming Units (SFU), displaying the absolute amount of cells producing a certain cytokine, and spot size, serving as measurable indicator for the average amount of a certain cytokine of all producing cells (larger spot size represents more cytokine being produced) using a ImmunoSpot^®^ Series 6 Alfa ELISpot Analyzer ENTRY (CTL, Shaker Heights, Ohio, USA). Samples were always run in duplicates and the mean of both values was used for data analysis. Scanning and counting parameters were optimized to obtain the best results and were kept constant throughout the whole study. All scans were manually quality controlled by a single person in our team.

### 2.6 Statistical analysis

Data was analyzed using GraphPad Prism version 10.4.0 (GraphPad Software, Boston, Massachusetts, USA) for statistical analysis. Insufficient sample volume, patient unavailability due to clinical intervention, measured values below or above detection level and equipment breakdown account for occasional differences in numbers for individual parameters. The dataset was tested for normality using the Shapiro-Wilk test. Differences between the descriptive measures of outcome groups were assessed using a One-way ANOVA. Relationships between categorical variables were assessed using fisher´s exact test. Correlation between continuous variables was assessed using Pearson´s correlation. Differences between the groups at individual timepoints and in comparison to the control population were assessed using the Mann-Whitney-U-Test. The threshold for significance was considered to be p < 0.05. Receiver Operating Characteristic (ROC) was calculated using the Wilson/Brown method.

## 3 Results

### 3.1 Patient demographics

34 Patients with an ISS ≥ 18 were prospectively enrolled in the study (**Table 1**), the median age was 50, with an Interquartile Range (IQR) of 32-72, with 27% (n=9) patients being female. The median ISS and New ISS were 26 (IQR 22-31) and 34 (IQR 27-42), respectively. When grouped by physical performative outcome (**Table 2**), the two groups showed no significant difference in injury severity (ISS, NISS) or in injury patterns (AIS), physiological parameters and most laboratory values on admission. Lactate dehydrogenase (LDH) levels were significantly higher in the group with favorable outcome (**Table 2**). Patients with a less favorable physical performative outcome were significantly older compared to favorable outcome, and had a significantly higher Charlson Comorbidity Index (CCI), a score which takes into account various comorbidities and estimates 10 year survival dependent on its value (34) (**Table 2**). When grouped by development of MODS (**Supplementary Table 1**), the groups did not show significant differences in overall injury severity, however the group that went on to develop MODS showed a higher AIS Face and a lower AIS Abdomen than the other group, although only by a median of 1 and 2 points respectively. The group developing MODS also had a significantly lower Glasgow Coma Scale (GCS). Laboratory values were not significantly different, but the group developing MODS was significantly older. When grouped by in-hospital mortality (**Supplementary Table 2**), there were no significant differences in injury severity, injury pattern or laboratory values either, however the non-survivors were significantly older and had a significantly decreased heart rate upon admission when compared to the survivors.

**Table 1:** Patient cohort versus control cohort.

Value	Whole -Cohort n=34 (Median + IQR)	Healthy Controls n=25 (Median + IQR)	p value of difference between groups
**Age**	**50** (32 - 72)	**46** (30-63)	0.5127
**Female**	**n=9** (26.5%)	**n=10** (40%)	0.3982
**CCI**	**1** (0 - 3)		
**With TBI**	**n=21** (61.8%)		
**ISS**	**26** (22 - 31)		
**NISS**	**34** (27-42)		
**AIS Head**	**3** (0 - 4)		
**AIS Face**	**0** (0 - 1)		
**AIS Chest**	**3** (0 -4)		
**AIS Abdomen**	**0** (0 - 2)		
**AIS Extremeties/Pelvis**	**2** (0 - 3)		
**AIS External**	**0** (0 - 0)		
**CK**	**291** (180 - 581)		
**LDH**	**350** (254 - 538)		
**Troponin T**	**12** (8.5 - 26)		
**Base Excess**	**-1.2** (-3.3 - 0.9)		
**Lactate**	**2** (1 - 3)		
**GCS**	**12** (5 - 15)		
**Heartrate**	**82** (71 - 97)		
**Systolic BP**	**120** (100 - 140)		
**Diastolic BP**	**77** (60 - 80)		

Descriptive measures and available laboratory values at admission of the overall patient cohort as well as the healthy control cohort (unless otherwise specified values represent the median (shown in bold) with interquartile range shown in brackets below; p values assessed with fisher´s exact test, bold values represent p values below 0.05; AIS, abbreviated injury scale; CCI, Charlson Comorbidity Index; BP, Blood Pressure in mmHg; CK, creatine kinase activity in U/l; GCS, Glasgow Coma Scale; ISS, Injury Severity Score; LDH, Lactate Dehydrogenase activity in U/l; Lactate in mmol/l; Troponin T in ng/l; NISS, New Injury Severity Score; TBI, traumatic brain injury).

**Table 2:** Favorable versus unfavorable physical performance cohort.

Value	Favourable Outcome n=15 (Median + IQR)	Unfavourable Outcome n=19 (Median + IQR)	p value of difference between groups
**Age**	**34** (24 - 51)	**64** (44 - 84)	**0.0007**
**Female**	**n=4** (26.7%)	**n=5** (26.3%)	> 0.9999
**CCI**	**0** (0 - 1)	**3** (0 - 6)	**0.0029**
**With TBI**	**n=9** (60.0%)	**n=12** (63.2%)	> 0.9999
**ISS**	**25** (19 - 29)	**29** (22 - 35)	0.2780
**NISS**	**27** (22-41)	**34** (29-43)	0.1247
**AIS Head**	**1** (0 - 3)	**3** (0 - 5)	0.1224
**AIS Face**	**0** (0 - 0)	**0** (0 - 2)	0.1395
**AIS Chest**	**3** (0 -4)	**3** (0 -4)	0.7318
**AIS Abdomen**	**0** (0 - 2)	**0** (0 - 1)	0.1763
**AIS Extremeties/Pelvis**	**3** (0 - 3)	**2** (1 - 3)	0.7139
**AIS External**	**0** (0 - 1)	**0** (0 - 0)	0.3096
**CK**	**323** (228 - 651)	**263** (153 - 533)	0.4149
**LDH**	**557** (252 - 667)	**333** (254 - 364)	**0.0095**
**Troponin T**	**10.5** (8 - 98)	**14** (9 - 21)	0.0849
**Base Excess**	**-0.9** (-3.3 - 1.4)	**-1.5** (-3.4 - 0.7)	0.6726
**Lactate**	**2.5** (1 - 3)	**2** (1 - 3)	0.3607
**GCS**	**14.5** (3 - 15)	**10** (5 - 14)	0.3211
**Heartrate**	**85** (80 - 103)	**78** (65 - 96)	0.1078
**Systolic BP**	**109** (100 - 140)	**120** (100 - 140)	0.6387
**Diastolic BP**	**75** (57 - 80)	**80** (70 - 90)	0.2603

Descriptive measures and available laboratory values at admission of the favorable and unfavorable physical performative outcome groups (unless otherwise specified values represent the median (shown in bold) with interquartile range shown in brackets below; p values between categorical variables assessed using fisher´s exact test; p values between continuous variables assessed with One-way ANOVA, bold values represent p values below 0.05; AIS, abbreviated injury scale; CCI, Charlson Comorbidity Index; BP, Blood Pressure in mmHg; CK, creatine kinase activity in U/l; GCS, Glasgow Coma Scale; ISS, Injury Severity Score; LDH, Lactate Dehydrogenase activity in U/l; Lactate in mmol/l; Troponin T in ng/l; NISS, New Injury Severity Score; TBI, traumatic brain injury).

### 3.2 Functional dynamic T cell-specific cytokine release

**Figure 1:**
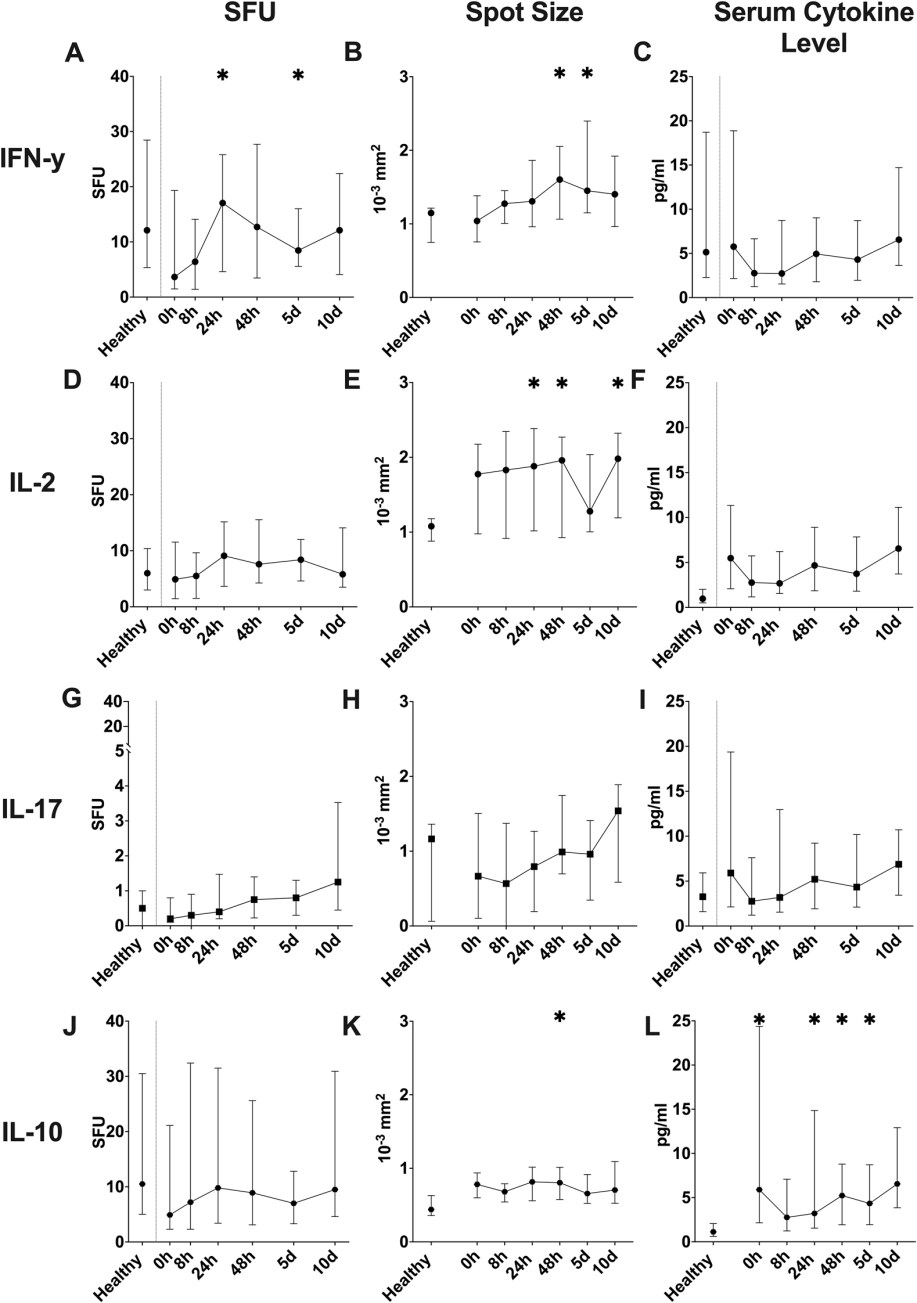
Overall dynamic of T cell-specific CD3/28-stimulated cytokine release in ELISpot assay **(A-L)**. Counted spot forming units (SFU) per 1µl of whole blood of IFN-γ **(A)**, IL-2 **(D)**, IL-17 **(G)**, and IL-10 **(J)** in absolute numbers. Mean of the size of all counted spots (Spot Size) for IFN-γ **(B)**, IL-2 **(E)**, IL-17 **(H)** and IL-10 **(K)** shown in 10^-3^ square millimeters. Serum cytokine concentrations for IFN-γ **(C)**, IL-2 **(F)**, IL-17 **(I)** and IL-10 **(L)** shown in pg/ml. (median ± interquartile range; * = p < 0.05 compared to healthy control population, Mann-Whitney-U-Test).

In comparison to the healthy controls, the trauma cohort showed a distinct release pattern of IFN-γ, IL-2, IL-17 and IL-10 in whole blood (**Figure 1**), when stimulated with anti-CD3/28-antibodies as a T cell-specific stimulus (35). IFN-γ SFU (**Figure 1**) trended to be decreased at the 0 and 8 hour timepoint and showed a slight increase at 24 hours compared to healthy controls. The spot size of the IFN-γ spots (**Figure 1**) increased steadily, reaching a significantly higher size at 48 hours and 5 days, then decreasing slightly. IL-2 SFU (**Figure 1**), while also showing a slight increase after 24 hours, did not differ significantly from the healthy controls at any timepoint. Spot size of the IL-2 spots (**Figure 1**) also showed a general upward trend, with significantly higher size at 24 and 48 hours as well as 10 days. IL-17 SFU (**Figure 1**) also did not differ significantly between the trauma cohort and the controls, however it should be noted that it showed a steady uptick of the median values over the 10-day study period, which however was non-significant. Spot size of the IL-17 spots (**Figure 1**) did not differ from the controls at any time but trended to increase constantly over the observed time period. IL-10 SFU (**Figure 1**) showed a general upward trend, while never differing significantly compared to the control cohort. Size of the IL-10 spots (**Figure 1**) remained relatively stable while constantly being non-significantly elevated throughout the study period, with the only significant increase at 48 hours compared to the control value. When evaluating systemic cytokine concentrations, IFN-γ (**Figure 1**), IL-2 (**Figure 1**) and IL-17 (**Figure 1**) concentrations showed a slight decrease at 8 and 24 hours with a general upward trend until day 10, while not showing any significant differences compared to the control cohort. Systemic IL-10 (**Figure 1**) concentrations showed a trend similar to the other studied cytokines, however IL-10 concentrations were significantly higher in the patient cohort than in the control cohort at 0, 24 and 48 hours as well as at day 5. When investigating the differences in the functional cytokine release depending on patient gender (**Supplementary Figure 9**) we could not reveal any significant differences between the genders at any given timepoint.

### 3.3 Dynamic systemic cytokine concentrations

**Figure 2:**
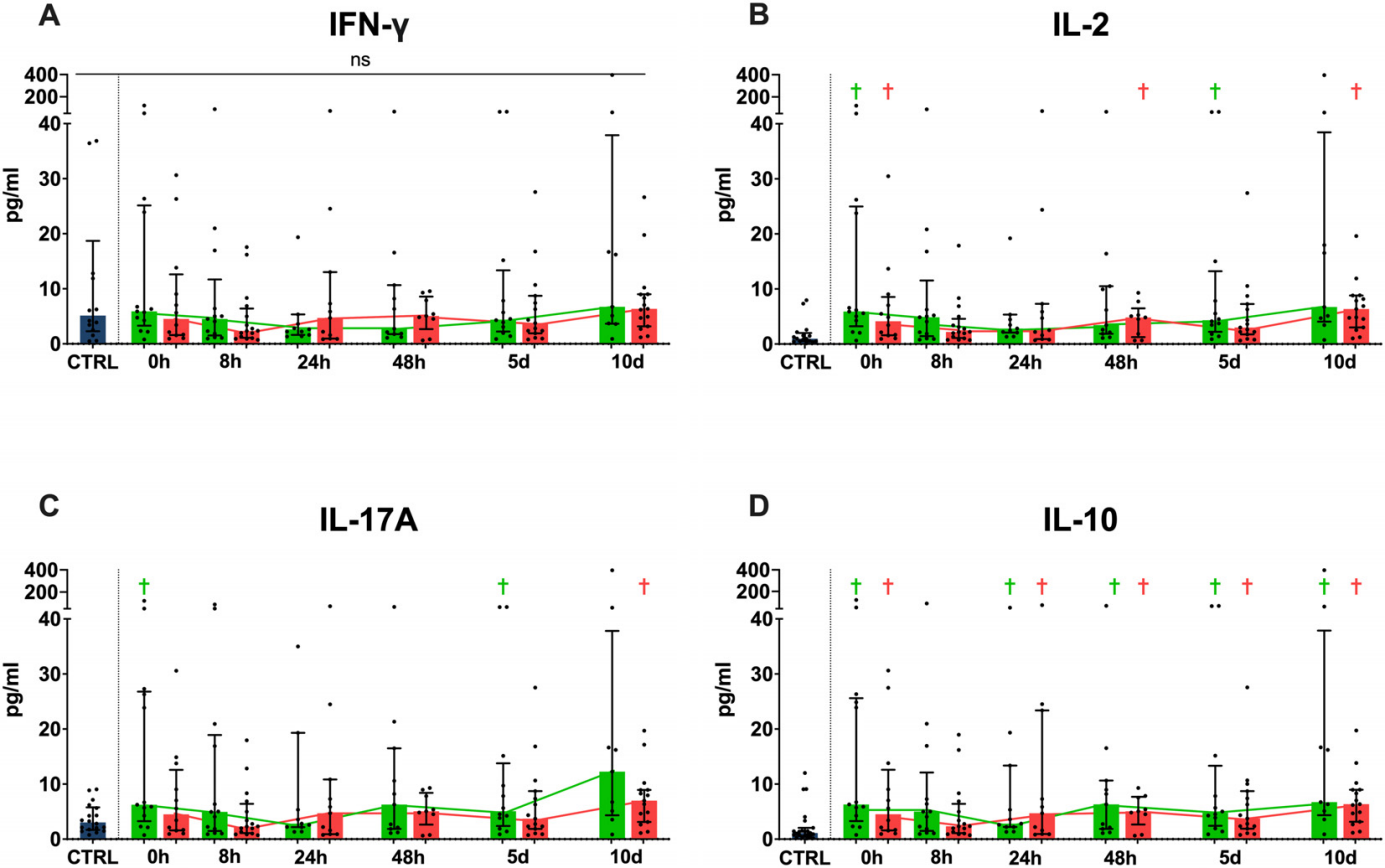
Cytokine concentrations in serum. Systemic IFN-γ **(A)**, IL-2 **(B)**, IL-17 **(C)** and IL-10 **(D)** concentrations shown in pg/ml. (Blue = healthy control population, green = favorable physical performative outcome, red= unfavorable physical performative outcome; † = p < 0.05 compared to healthy control population, ns = no significant differences between any timepoints or compared to healthy controls; Mann-Whitney-U-Test; median ± interquartile range).

Serum cytokine analysis (**Figure 2**) yielded differing results for each studied cytokine. For systemic IFN-γ concentrations (**Figure 2**), we did not see any significant differences either between the two outcome groups or compared to the controls at any timepoint. IL-2 (**Figure 2**) concentrations showed higher values for the favorable group at 0 hours and 5 days, while the unfavorable group had significantly higher concentrations at 0 and 48 hours as well as 10 days when compared to the healthy controls. There were no differences between the two outcome groups at any timepoint for IL-2 concentrations. IL-17A concentrations (**Figure 2**) in the favorable outcome group were significantly higher compared to the control cohort at 0 hours and 5 days, while the unfavorable group only showed higher concentrations than the controls at the 10-day timepoint. IL-17A concentrations did not differ significantly between the two outcome groups at any timepoint. IL-10 concentrations (**Figure 2**) showed significantly higher serum concentrations in both groups compared to the healthy control subjects at all timepoints except at 8 hours, however, it did not show differing concentrations between the two outcome groups.

When grouped by development of MODS (**Supplementary Figure 1**), we see significantly higher systemic concentrations of IFN-γ, IL-2 and IL-10 at 24 hours in the group that developed MODS during their hospital stay, while IL-17A did not differ significantly between the two groups. Additionally, there are various differences when comparing either group with the healthy control cohort (**Supplementary Figure 1**). Grouping by survival vs. in hospital mortality (**Supplementary Figure 2**) did not reveal any significant differences between the two groups at any timepoint for any of the studied cytokines, but did show several differences when comparing either group with the control cohort (**Supplementary Figure 2**).

### 3.4 Outcome prediction using functional immunomonitoring

We compared the patient’s outcome depending on their physical performative outcome between each other at each timepoint regarding their SFU and the respective average size of these spots. This was conducted after T cell-specific CD3/28 stimulation for different cytokines in the ELISpot assay in order to characterize dynamic T cell functionality and find differences that may be used for predictive purposes.

**Figure 3:**
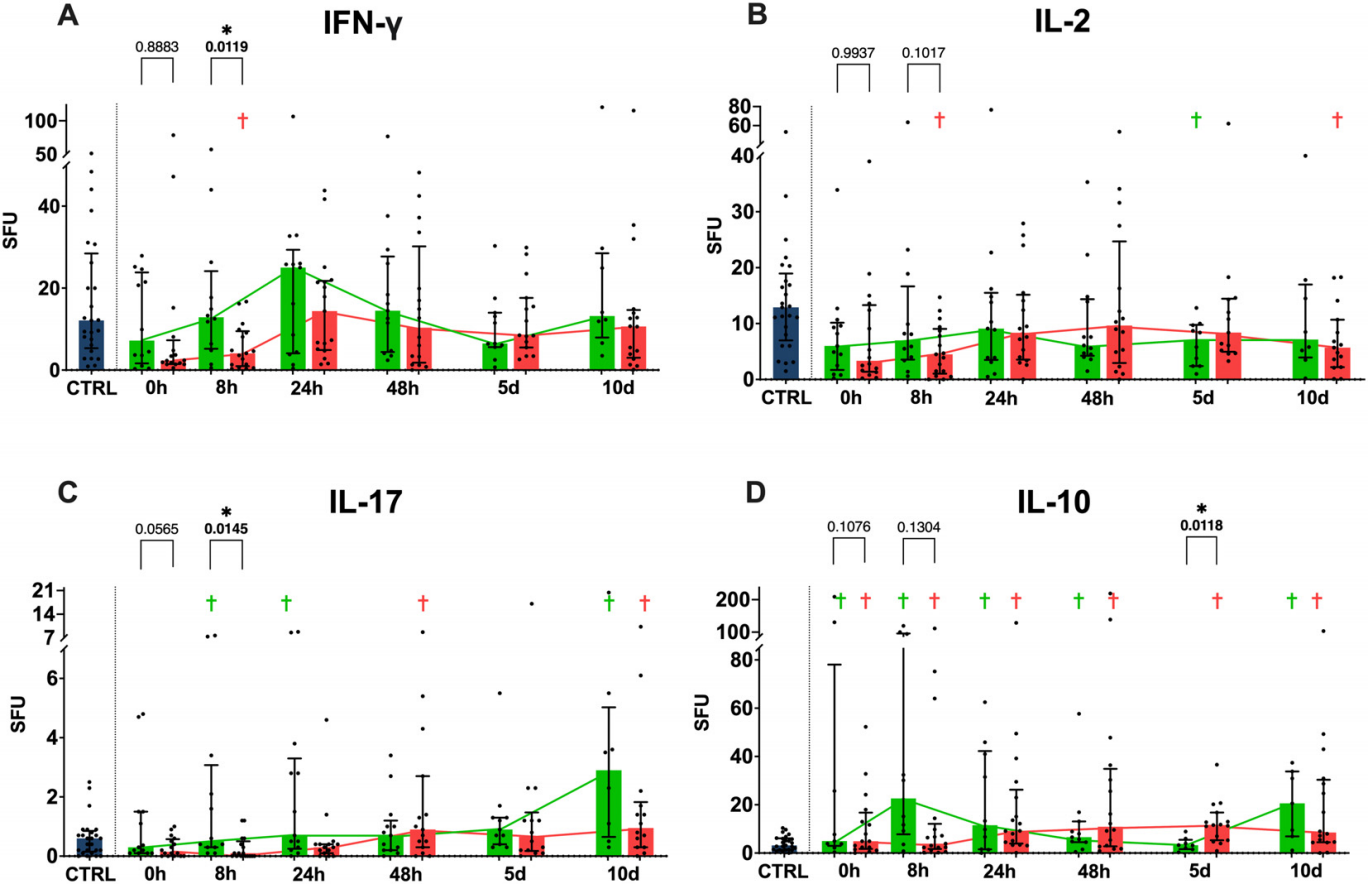
Functional cytokine release in ELISpot. Anti CD3/28 stimulated ELISpot spot forming units (SFU) per 1µl in whole blood for IFN-γ **(A)**, IL-2 **(B)**, IL-17 **(C)** and IL-10 **(D)** shown in absolute numbers. p values between the two outcome groups shown for all 0 and 8h timepoints. (Blue = healthy control population, green = favorable physical performative outcome, red= unfavorable physical performative outcome; † = p < 0.05 compared to healthy control population; * = p < 0.05 between the two outcome groups at the timepoint; Mann-Whitney-U-Test; median ± interquartile range).

When examining functional IFN-γ release, we see significantly higher SFUs in the favorable physical performative outcome group compared to the unfavorable group at the 8 hour timepoint, as well as a lower SFUs in the unfavorable physical performative outcome cohort compared to the healthy controls at 8 hours (**Figure 3**). IL-2 SFUs were lowered for the unfavorable outcome group at 8 hours and 10 days compared to the healthy population, while showing no significant differences between the two outcome groups at any timepoint (**Figure 3**). IL-17 SFUs were significantly higher in the favorable physical performative outcome group compared to the unfavorable one at 8 hours. Additionally, the IL-17 SFUs in the favorable outcome group exceeded that of the control cohort at 8 and 24 hours and 10 days and the unfavorable group at 5 and 10 days (**Figure 3**). The group with favorable physical performative outcome had significantly higher IL-10 SFUs upon admission, at 8, 24 and 48 hours and at 10 days, while the unfavorable outcome group had higher IL-10 SFUs at all measurement timepoints when compared to the control cohort. Additionally, IL-10 SFUs were significantly higher in the unfavorable outcome group at the 5-day timepoint compared to the favorable outcome group (**Figure 3**).

**Figure 4:**
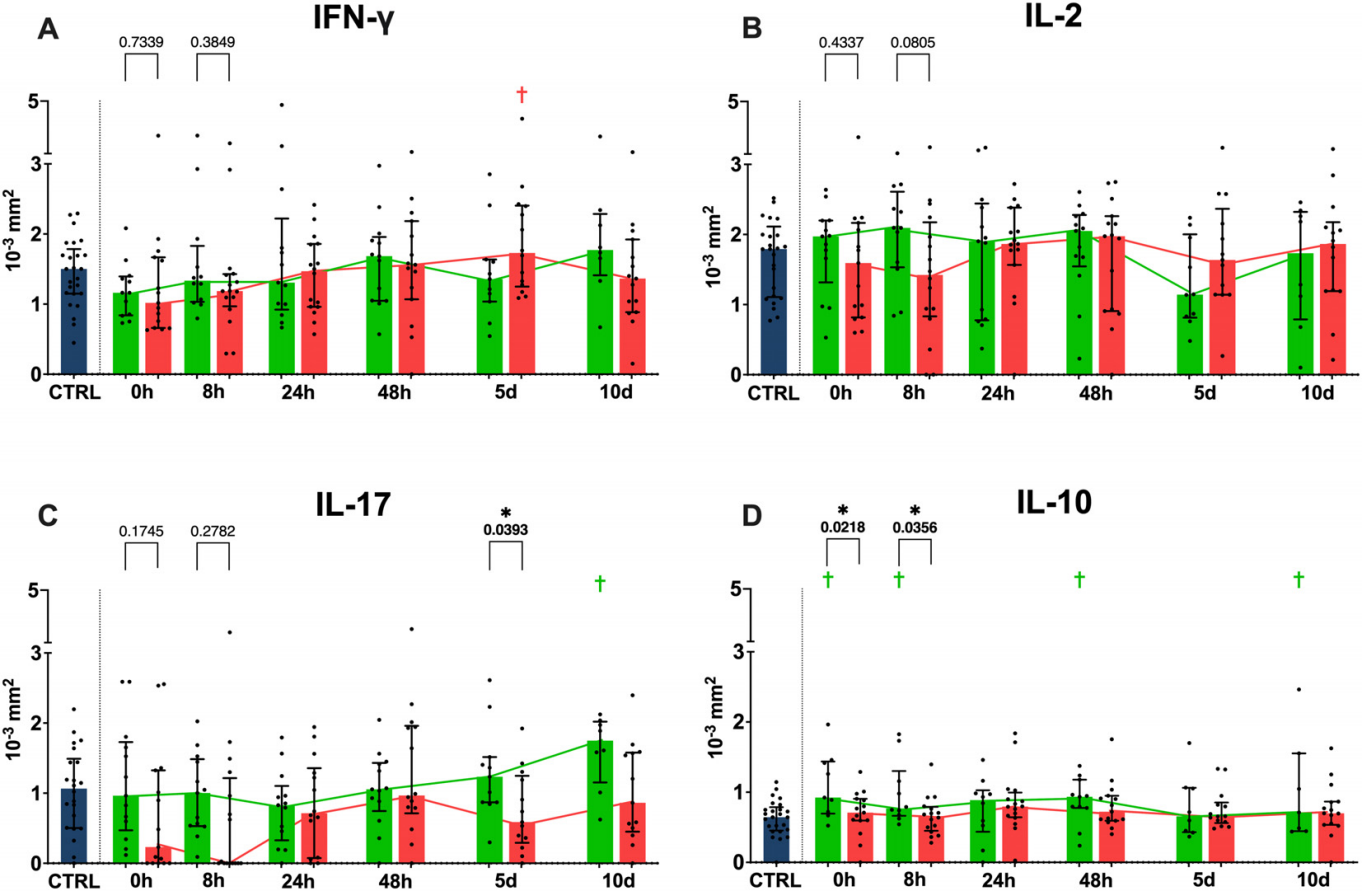
Functional cytokine release in ELISpot. Anti CD3/28 stimulated ELISpot spot size for IFN-γ **(A)**, IL-2 **(B)**, IL-17 **(C)** and IL-10 **(D)** shown in 10^-3^mm^2^. p values between the two outcome groups shown for all 0 and 8h timepoints. (Blue = healthy control population, green = favorable physical performative outcome, red= unfavorable physical performative outcome; † = p < 0.05 compared to healthy control population; * = p < 0.05 between the two outcome groups at the timepoint; Mann-Whitney-U-Test; median ± interquartile range).

The comparison for physical performative outcome regarding IFN-γ spot size (**Figure 4**) revealed a higher spot size for the unfavorable physical performative outcome group at 5 days when compared to the healthy controls, while showing no significant differences between the two outcome groups. IL-2 spot size did not differ either between the two outcome groups or compared to the healthy controls at any timepoint (**Figure 4**). IL-17 spot size was higher for the favorable physical performative outcome group at 5 days compared to the unfavorable group, with the favorable group also showing a higher IL-17 spot size compared to the control cohort at 10 days (**Figure 4**). IL-10 spot size was significantly higher in the favorable physical performative outcome group compared to the unfavorable one both at 0 and 8 hours, while the favorable physical performative outcome group also had higher spot size compared to the controls at 0, 8 and 48 hours (**Figure 4**).

When compared by development of MODS during the patient’s hospital stay, we did not see any significant differences between our two outcome groups regarding SFUs, however, the SFUs were partially significantly different when comparing the two groups to the healthy control cohort (**Supplementary Figure 3**). The comparison by MODS development regarding spot size (**Supplementary Figure 4**) revealed a significantly higher average spot size in the group without MODS development for IFN-γ at 10 days, for IL-2 at 8 hours, and IL-17 at 0 hours and 5 days, with IL-10 showing no difference between the two outcome groups. IFN-γ, IL-17 and IL-10 also showed several differences between the control cohort and either the group with or without MODS (**Supplementary Figure 4**).

When grouped by in-hospital mortality, the non-surviving cohort showed higher IL-2 SFUs at 5 days (**Supplementary Figure 5**). All other timepoints and cytokines revealed no difference between the two outcome groups (**Supplementary Figure 5**). There were several differences between the survivors and non-survivors and the healthy control cohort (**Supplementary Figures 5**, **6**).

ROC analysis for the 8 hour timepoint (**Supplementary Figure 7**) regarding spot forming units and physical performative outcome reveals an area under curve (AUC) of 0.7721 (p=0.0140) for IFN-γ (**Supplementary Figure 7**), of 0.8137 (p=0.0046) for IL-17 (**Supplementary Figure 7**) and of 0.7471 (p=0.0350) for IL-10 (**Supplementary Figure 7**), with the ROC analysis for IL-2 (**Supplementary Figure 7**) not displaying statistically significant results (p=0.2400).

### 3.5 Quantitative dynamics of T cell subtypes

**Figure 5:**
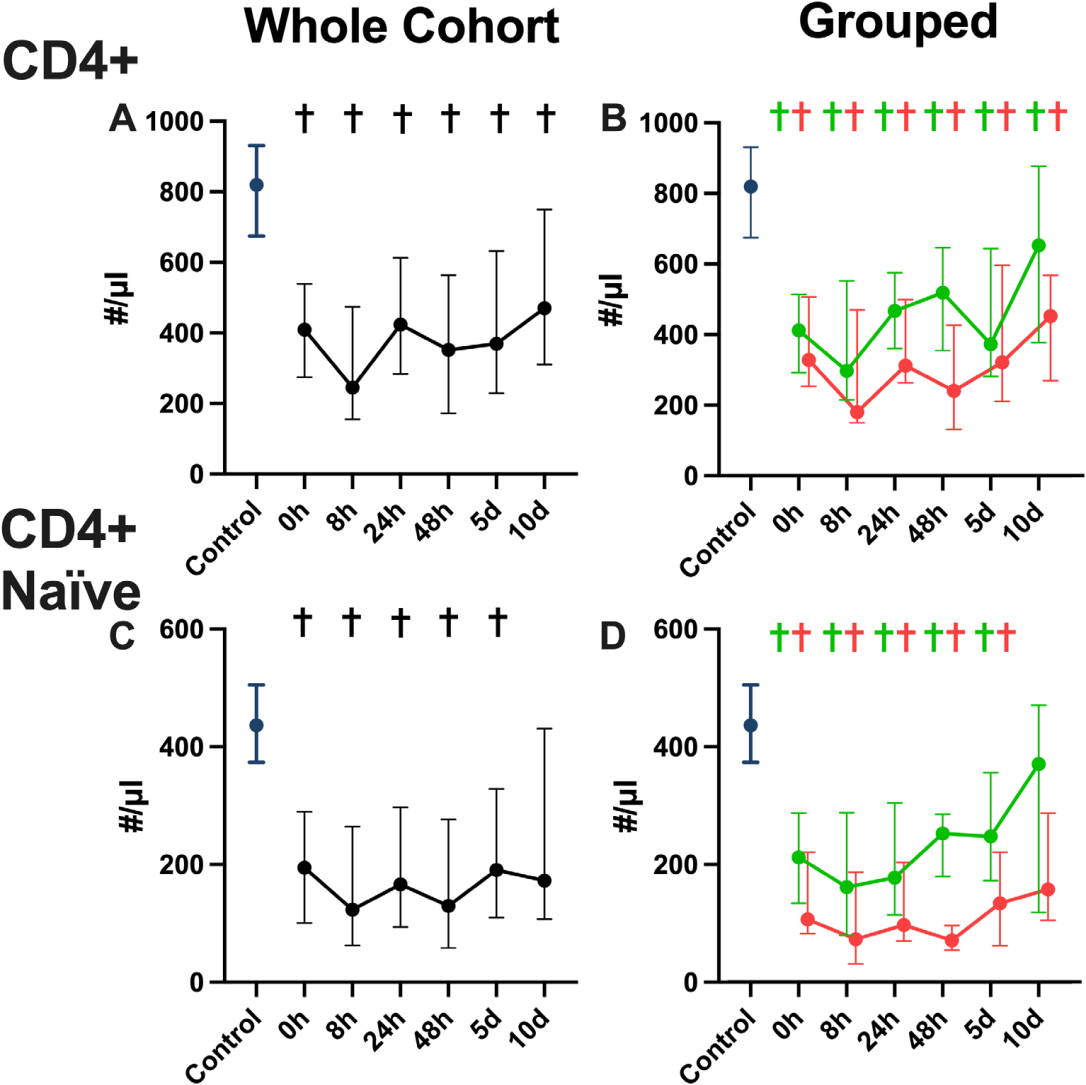
CD4 T cell numbers in whole blood. CD4 helper T cell numbers for the whole patient cohort **(A)** the favorable and unfavorable physical performative outcome groups **(B)**, as well as CD4 naïve (CD45Ra+ CCR7+) helper T cell numbers for the whole patient cohort **(C)** the favorable and unfavorable physical performative outcome groups **(D)** shown in absolute number per μl of whole blood. (Blue = healthy control population, green = favorable physical performative outcome, red= unfavorable physical performative outcome; * = p < 0.05 between the two outcome groups; † = p < 0.05 compared to healthy control population; Mann-Whitney-U-Test; median ± interquartile range).

**Figure 6:**
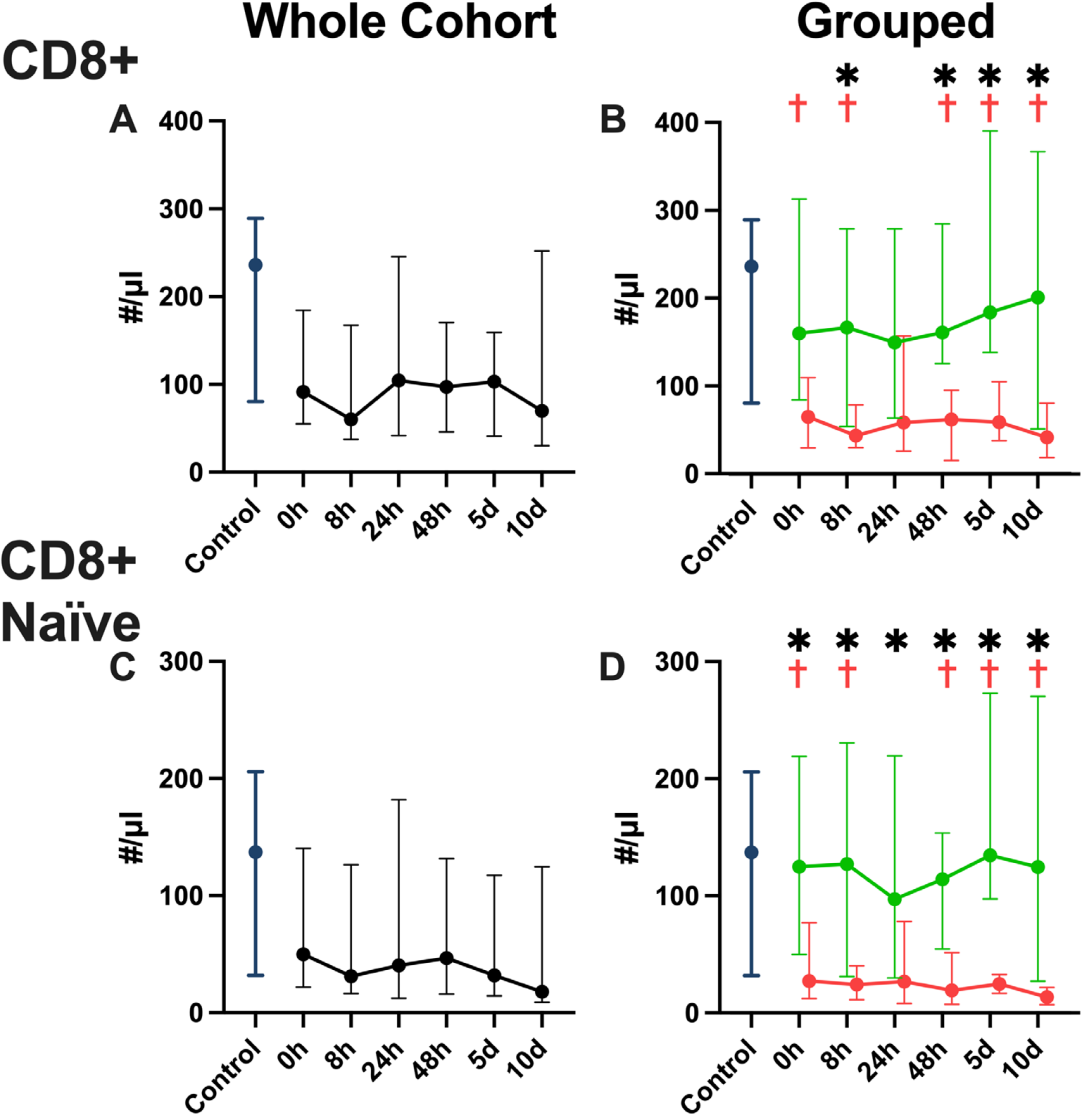
CD8 T cell numbers in whole blood. CD8 cytotoxic T cell numbers for the whole patient cohort **(A)** the favorable and unfavorable physical performative outcome groups **(B)**, as well as CD8 naïve (CD45Ra+ CCR7+) cytotoxic T cell numbers for the whole patient cohort **(C)** the favorable and unfavorable physical performative outcome groups **(D)** shown in absolute number per μl of whole blood. (Blue = healthy control population, green = favorable physical performative outcome, red= unfavorable physical performative outcome; * = p < 0.05 between the two outcome groups; † = p < 0.05 compared to healthy control population; Mann-Whitney-U-Test; median ± interquartile range).

We found a significant decrease of CD4 T cell numbers (**Figure 5**) in the overall cohort when compared to the healthy controls over all timepoints. When examining the favorable physical performative outcome group (**Figure 5**) and the unfavorable physical performative outcome group (**Figure 5**) separately, we see decreased CD4 T cell numbers compared to the controls and, while a continuous upward trend is noted comparing 0 hours and day 10, only the group with favorable outcome reaches the same level as the control cohort at the 10 day timepoint. When examining naïve CD45Ra+ CCR7+ CD4 T cells (36) in the overall cohort (**Figure 5**), we found significantly decreased numbers at all timepoints, except at 10 days compared to healthy controls. Naïve CD4 T cells were significantly decreased compared to the healthy control cohort at all timepoints except at 10 days in both the favorable (**Figure 5**) and unfavorable physical performative outcome group (**Figure 5**). When examining CD8 T cell numbers we see lower median values for the overall patient cohort (**Figure 6**) compared to the controls, without significant differences. In the favorable physical performative outcome group we see no significant differences compared to the controls when examining CD8 T cell numbers (**Figure 6**). The unfavorable physical performative outcome group, however, had significantly lower CD8 T cell numbers at all timepoints except 0 and 24 hours (**Figure 6**). Naïve CD8 T cell numbers did not differ significantly compared to the control cohort in both the overall patient cohort (**Figure 6**) as well as the favorable physical performative outcome group (**Figure 6**). In the unfavorable physical performative outcome group, however, we see significantly decreased numbers of naïve CD8 T cells at alle timepoints except at 24 hours (**Figure 6**)

### 3.6 Correlation of IFN--γ release and CD8 T cell numbers

**Figure 7:**
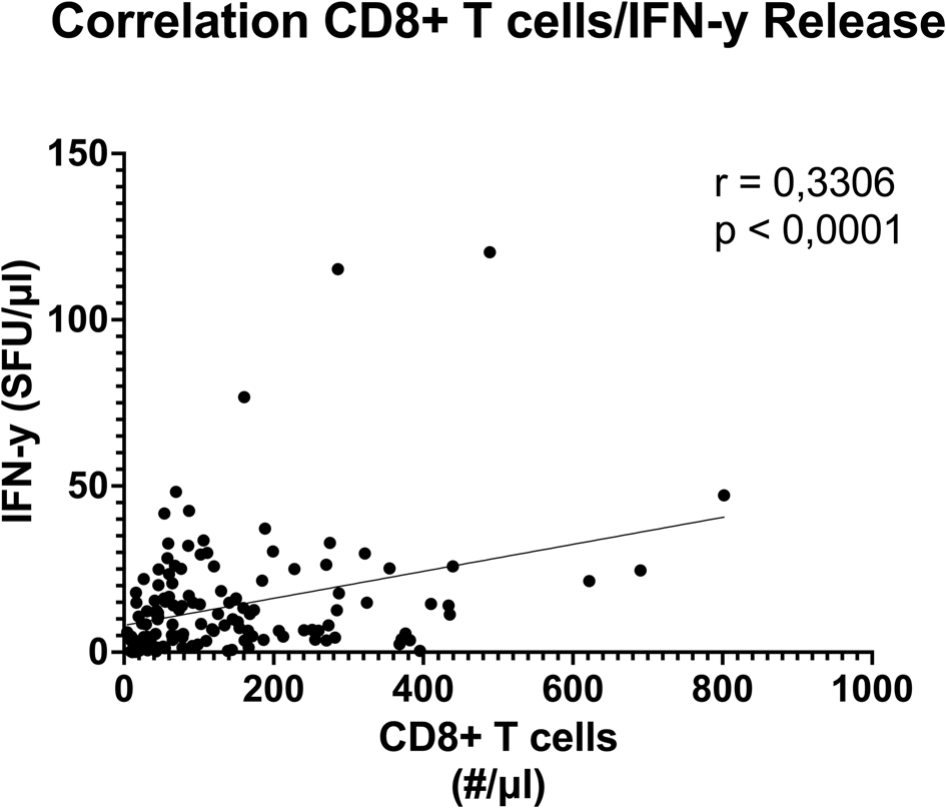
Correlation between blood CD8 T cell numbers and functional anti CD3/28 stimulated IFN-γ release. Result of Pearson´s correlation test between whole blood CD8 T cells per μl and IFN-γ spot forming units (SFU) over all studied timepoints show moderate, significant (r=0.3306) correlation. (Threshold for significance p < 0.05).

The correlation analysis of CD8 T cell numbers and IFN-γ SFU over all timepoints (**Figure 7**) revealed a statistically significant positive correlation (p <0.0001, r= 0.3306).

### 3.7 Ex-vivo stimulation with IL-7 increases functional IFN-γ release

**Figure 8:**
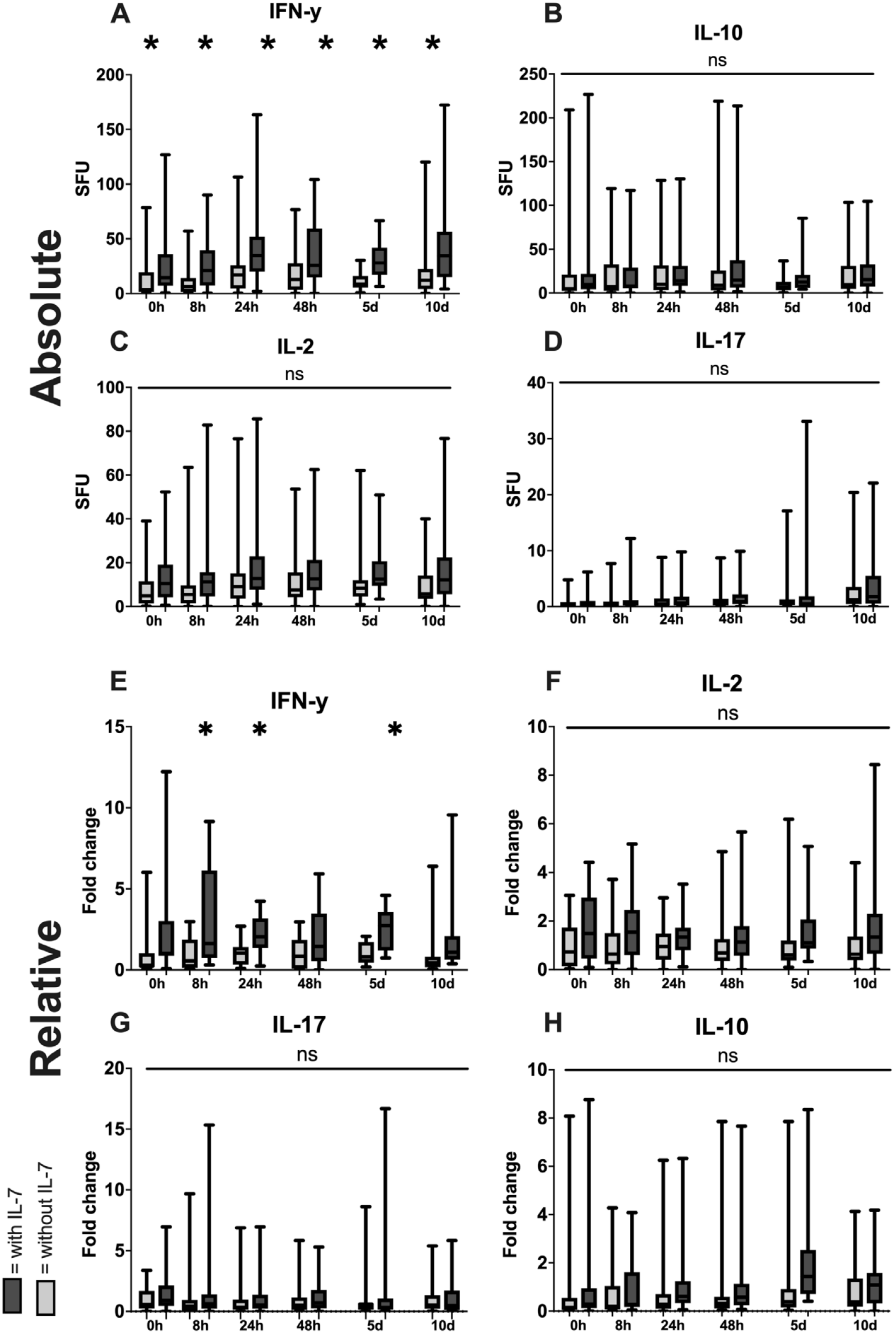
Increase of functional cytokine release through addition of IL-7. Absolute increase in number of spot forming units (SFU) per 1µl of whole blood with anti CD3/28 antibodies with IL-7 compared to only anti CD3/28 stimulation for IFN-γ **(A)**, IL-2 **(B)**, IL-17 **(C)** and IL-10 **(D)** as well as relative increase in SFU by the addition of IL-7 (All values divided by the mean of the CD3/28 stimulated group at the respective timepoint) for IFN-γ **(E)**, IL-2 **(F)**, IL-17 **(G)** and IL-10 **(H)**. (White = stimulated with anti CD3/28 antibodies, grey = stimulated with anti CD3/28 antibodies in combination with IL-7; * = p < 0.05 between the two simulation methods; Mann-Whitney-U-Test; median ± interquartile range).

When the whole blood was co-incubated with both anti-CD3/28 as well as recombinant IL-7 (**Figure 8**) we found a significant overall rise in IFN-γ SFU at all timepoints when compared with anti-CD3/28 stimulation alone (**Figure 8**). IL-2, IL-10 and IL-17 SFU (**Figure 8B**) were not significantly affected by coincubation with IL-7. Next to absolute SFUs per 1 µl whole blood, all counts were divided by mean of the only CD3/28 stimulated values at the respective timepoint in order to assess the relative change in SFU in response to IL-7 (**Figure 8E**). We found a significant increase in relative IFN-γ production with IL-7 at 8 and 24 hours as well as at 5 days. (**Figure 8**). All other cytokines measured showed no significant differences in response to IL-7 stimulation (**Figure 8F**)

## 4 Discussion

### 4.1 Key findings

The dynamic functional immunomonitoring of T cells in polytraumatized patients reveals an early significant reduction of stimulated T cell-specific IFN-γ and IL-17 release (**Figure 3**), as well as a trend towards a non-significant decrease of IL-2 and IL-10 (**Figure 3**) in patients with unfavorable physical performative outcome. MODS development is also correlated with decreased IL-2 (8 hours) and IL-17 (on admission) production early post-trauma (**Supplementary Figure 4**). This leads to the conclusion that T cell function of all Th cell types, inflammatory and immunosuppressive, is impaired already on admission and correlates with adverse outcome. Moreover, the reduction of CD8 (**Figure 5**) and more prominently naïve CD8 T cells (**Figure 5**) correlated with adverse outcome (**Figure 5**) and seem causal for the systemic impairment of functional IFN-γ release (**Figure 7**). This is of note as we found functional IFN-γ release to be predictive for adverse outcome (**Supplementary Figure 7**). The ex vivo application of IL-7 proved to be specifically increasing IFN-γ production (**Figure 8**) and might suit as an early interventional drug to improve adaptive function in polytraumatized patients and therewith potentially improve outcome.

### 4.2 Characteristic suppressed overall T cell functionality post trauma

The measured cytokine release occurred after T cell-specific stimulation with anti CD3/28 antibodies, which means that our measurement of different cytokines can be attributed specifically to T cells (37). IFN-γ is a pro-inflammatory cytokine with anti-viral, anti-tumor and immune modulatory effects (38). IL-2 is an expansion factor for all types of activated T cells (39). IL-17 is a pro-inflammatory cytokine which, among other functions, induces other signaling molecules in order to recruit immune cells (40). IL-10 has predominantly anti-inflammatory effects and causes downregulation of Th1 cytokines and MHC class II antigens among other things (41). IFN-γ and IL-2 release is attributed to Th1 cells, with IL-2 also being produced by CD8 T cells (42), while IL-17 production is characteristic for Th17 cells (43). IL-10 is mainly produced by regulatory T cells (Tregs) (44). Using ELISpot as an immunomonitoring method we were able to provide the total amount of cells producing a certain cytokine, reflected by SFU and the relative amount per cell, reflected by the spot size. Our data indicates an initial functional suppression of Th1 cell activity in the overall trauma cohort in comparison to the healthy control subjects, with Th1 being a significant IFN-γ producer (45). IFN-γ reverses at the 24-hour mark where we see a peak IFN-γ release, after which it returns to a baseline activity. This is remarkable since it is has been postulated that change in T cell function takes place over longer time periods, with naïve T cells for example taking up to 20 hours to commit to proliferation (46) and T cell activation taking up to 8 hours (47). The mechanism of this early functional depression is unknown. Our data indicates that especially the impaired function of inflammatory Th1 and Th17 cells (adverse physical performative outcome for IFN-γ at 8 hours (**Figure 3A**) and development of MODS for IL-17 on admission (**Supplementary Figure 3**) renders the patient susceptible to improper recovery. However the impairment of Treg function [trend to decreased IL-10 release at 8 hours correlating with unfavorable physical performative outcome (**Figure 3**)] also correlates with adverse outcome. We believe the suppression of both pro-inflammatory and immunosuppressive T cell-subsets is what causes adverse outcomes in trauma patients (unfavorable physical performative outcome and MODS) rather than the postulated switch from a predominantly Th1 to Th2 adaptive immune response (16). Our results do not reveal the cause of that suppressive characteristic. A possible explanation could be initial overwhelming activation of T cells by danger-associated molecular patterns (DAMPs), resulting in loss of function or apoptosis (13, 48). Th17 cells of the whole cohort show a steady increase in functional IL-17 release over the study period, while IL-10 release remains relatively stable. When investigating gender specific differences in the post trauma immune response there were no significant differences in functional cytokine release when comparing the male and female patient cohort with each other at any studied timepoint, suggesting that the post trauma functional cytokine release of T cells might be independent from gender (**Supplementary Figure 9**).

### 4.3 Balanced T cell functionality post trauma and early outcome prediction

The measured serum cytokine concentrations did not differ significantly between our outcome groups at any timepoint for any of the cytokines studied. This highlights the fact that systemic cytokine concentrations are not suitable to use as prognostic markers for physical performative outcome, and that functional measurements should be considered instead. Other studies investigating outcome prediction in trauma did not find clinically applicable prognostic value in serum concentrations of either IL-17 (49) or IL-10 (50). It seems like it depends which outcome should be predicted as we previously did use systemic IL-10 concentrations to predict sepsis development in trauma patients (51).

When grouped by performative outcome, the analysis of functional cytokine release shows that early functional IFN- γ production is associated with positive outcome, which might be due to its ability to recruit monocytes to the sites of injury or because of increased resistance to bacterial infections (38). Similarly, IL-17 shows an early increase with a steady rise in the group with favorable outcome. What positive effect IL-17 could have in the later stages is unclear. Both findings suggest that a strong inflammatory response by different T cell subsets in the first 24 hours after severe trauma has beneficial effects regarding patient outcome. IL-10 is trending higher during the first 24 hours in the group with favorable outcome, after which the unfavorable group shows significantly higher IL-10 levels at the 5 day timepoint. This suggests that IL-10, with its anti-inflammatory properties (52), is initially beneficial, along with the pro-inflammatory response [IFN-γ and IL-17 (**Figure 3**)] most likely balancing the immune response. However, once this anti-inflammatory response is no longer needed later in the course of illness, overproduction of IL-10 might be detrimental to the patients. A similar effect has been shown in burn injured and trauma patients, where higher systemic IL-10 levels were associated with the development of sepsis (51, 53). The different cytokines we measured show a similar and consistent overall trend between the outcome groups, which is surprising, considering that physical performative outcome is a broadly defined category in a small group of patients.

The measured differences in functional cytokine release showed promise for use as a predictive method with ROC curves revealing an area under curve (AUC) of 0.7721 (p=0.0140) for IFN-γ, 0.8137 for IL-17 (p=0.0046) and 0.7471 (p=0.0350) for IL-10 (**Supplementary Figure 7**). This shows that functional immunomonitoring could, if these findings are further validated, provide valuable, objective markers to identify patients at risk of adverse outcomes early during the course of their hospital stay, possibly at admission or during the first 8 hours, as demonstrated in our results. This could allow clinicians to administer more personalized care for these patients, possibly being able to prevent or ameliorate outcomes in these high risk patients.

### 4.4 Correlation of functional IFN-γ production and CD8 T cells

A decrease in circulating lymphocytes has been shown to be associated with adverse outcomes in trauma (7, 8). We also see decreased counts in our patient cohort, with CD4 T cells being decreased at all timepoints regardless of outcome, and CD8 T cells decreased only in the cohort with negative physical performative outcome. Especially a decrease in naïve CD8 T cells is associated with adverse outcomes, which is in line with previously published studies (54, 55). The negative effect of lower CD8 cell numbers in general could be attributed to a recently discovered effect of those cells on wound healing (56) or because of reduced capacity to fight of viral infections after trauma, however further research is needed to establish the exact mechanism and the causal relationship of these findings. The correlation revealed between CD8 T cell count and functional IFN-γ production has been shown before in a mixed cohort of ICU patients by Haem et al. (25), however the correlation was not yet proven for a trauma cohort. These findings show that, in trauma patients, a significant part of IFN-γ production can likely be attributed to CD8 T cells, which, with regard to our finding that IFN-γ could be beneficial, as well as some successful attempts to use IFN-γ as immune therapy in trauma (57), might explain the worse outcome of patients with reduced CD8 T cells. Further research needs to examine the proportion of different IFN-γ producing CD4 and CD8 T cells in trauma in order to prove causality.

### 4.5 IL-7 reverses T cell dysfunction

There are studies ongoing that investigate various immune modulatory therapies in patients with trauma or sepsis, with IL-7 being tested in one of these trials (ClinicalTrials.gov ID: NCT02640807). These trials focus on either granulocyte macrophage colony-stimulating factor or IL-7 to restore immune function in patients after polytrauma or in septic patients. IL-7 specifically has been shown to increase lymphocyte counts in septic patients (58) and has shown promising results in treating wound infections after trauma (29). IL-7 treatment has been shown to restore both CD4 and CD8 T cell counts in a randomized controlled trial in septic patients (58) as well as in animal models of sepsis (59). Patients treated with IL-7 in clinical trials for various disorders, including sepsis, cancer or acquired immune deficiency syndrome, consistently showed increases in circulating lymphocyte count, while showing little adverse effects, with a rash at the injection site being the only reported serious adverse effect. Importantly, serious immunological complications of IL-7 administration, like cytokine release syndrome or excessive immune activation, have not been observed in any of the patients (58, 60, 61). We showed that an ex vivo stimulation of T cells with IL-7 increases IFN-γ count specifically and does not significantly affect IL-2, IL-17 or IL-10 counts. Since higher early functional IFN-γ production seems beneficial in trauma, and IFN-γ administration has shown potential as a therapeutic in sepsis (62), we postulate that IL-7 selectively stimulates T cells that influence outcome positively, and that an early (possibly on admission) administration of IL-7 might prove to be a useful treatment for prevention of adverse outcome such as decreased physical performative outcome or MODS in polytraumatized patients.

### 4.6 Study limitations

This study has several limitations. The limited sample size and the fact that trauma is a very heterogeneous disease process. Because of the limited sample size, sub-group analyses for patients presenting either with or without traumatic brain injury (TBI), which is known to have an immune modulatory effect on its own (63), yielded no significant results due to the low number of patients without TBI (data not shown). Moreover, there is a difference in LDH concentration in the favorable and non-favorable physical performative outcome group with LDH being increased in the beneficial group (**Table 2**). It remains speculative to why that is, previous studies have shown organ-specific elevations in LDH after trauma (64, 65), and a larger study is needed to establish the cause of these results. Additionally, grouping patients depending on age (older or younger than 60 years) and beneficial or adverse outcome lacks significant results due to small subgroups (data not shown). However, we could show that the correlation of functional IFN-γ production and systemic CD8 T cell numbers correlate in both age groups (**Supplementary Figure 8**), indicating this effect is not age dependent. However, since our respective outcome groups all show significant differences in their age distribution, as well as differences in their CCI, it is possible that some of the difference we see in immune function could be either related to patient age, or their various preexisting conditions, and are not directly causally linked to outcomes.

## 5 Conclusion

In conclusion we demonstrated a distinct pattern of CD4 and CD8 T cells after trauma, both in the overall numbers and specifically in the naïve subpopulation, that differs significantly between groups with favorable and unfavorable outcome and showed how early IFN-γ and IL-17 production is likely beneficial in trauma patients, while late IL-10 production may have negative effects on outcome. Additionally, we showed how a significant portion if IFN-γ production can likely be attributed to CD8 T cells and that IL-7 may be a potent therapy to combat trauma induced immune suppression.

## Data Availability

The raw data supporting the conclusions of this article will be made available by the authors, without undue reservation.
