# Supplementary material for: Dynamic functional assessment of T cells reveals an early suppression correlating with adverse outcome in polytraumatized patients
Source: Front Immunol. 2025 Mar 24;16:1538516. doi: 10.3389/fimmu.2025.1538516 (PMC11973370; doi:10.3389/fimmu.2025.1538516)
Supplement: Supplementary file 1 [file DataSheet1.pdf]

Supplementary Material

Value	No MODS n=16 (Median + IQR)	MODS n=18 (Median + IQR)	p value of difference between groups
Age	40 (24 - 53)	63 (37 - 83)	**0.0162**
Female	n=6 (37.5 %)	n=3 (16.7 %)	0.2497
With TBI	n=8 (50.0 %)	n=13 (72.2 %)	0.2906
ISS	26 (22 - 29)	29 (22 - 39)	0.1536
NISS	27 (23-39)	36 (29-43)	0.0570
AIS Head	2 (0 - 4)	3 (2 - 5)	0.1427
AIS Face	0 (0 - 0)	1 (0 - 2)	**0.0116**
AIS Chest	3 (0 -4)	3 (0 -4)	0.7382
AIS Abdomen	2 (0 - 2)	0 (0 - 0)	**0.0279**
AIS Extremeties/Pelvis	3 (0 - 3)	2 (2 - 3)	0.8160
AIS External	0 (0 - 0)	0 (0 - 0)	0.8783
CK	305 (207 - 510)	264 (152 - 729)	0.7345
LDH	361 (268 - 635)	320 (249 - 427)	0.0850
Troponin T	10 (6 - 24)	16 (10 - 32)	0.7371
Base Excess	0.3 (-3.0 - 1.7)	-2 (-4 - 0.4)	0.0513
Lactate	1.8 (1.4 - 3.2)	1.8 (0.8 - 3.3)	0.8200
GCS	15 (9.5 - 15)	7 (4 - 13)	**0.0320**
Heartrate	84 (80 - 94)	80 (65 - 102)	0.8059
Systolic BP	123 (100 - 140)	120 (105 - 140)	0.7381
Diastolic BP	78 (60 - 80)	74 (67 - 84)	0.9976

Supplementary Table 1: Patient cohorts developing MODS versus those without MODS. Descriptive measures and available laboratory values at admission of the outcome groups that

developed MODS during their hospital stay and the group that did not. (unless otherwise specified values represent the median with interquartile range shown in brackets; p values between categorical variables assessed using fisher´s exact test; p values between continuous variables assessed with One-way ANOVA; TBI= traumatic brain injury; AIS = abbreviated injury scale; ISS = Injury Severity Score; NISS = New Injury Severity Score; CK = creatine kinase activity in U/l; LDH = Lactate Dehydrogenase activity in U/l; Lactate in mmol/l; Troponin T in ng/l; GCS= Glasgow Coma Scale; BP = Blood Pressure in mmHg)

Value	Survivors n=28 (Median + IQR)	Non Survivors n=6 (Median + IQR)	p value of difference between groups
Age	45 (26.5 - 60.5)	75.5 (48 - 88)	**0.0255**
Female	n=8 (28.5 %)	n=1 (16.7 %)	> 0.9999
With TBI	n=18 (64.3 %)	n=3 (50.0 %)	0.6529
ISS	26 (22 - 30)	27.5 (23 - 36)	0.7861
NISS	34 (25-41)	38.5 (29-52)	0.1353
AIS Head	3 (0 - 4)	1.5 (0 - 5)	0.7708
AIS Face	0 (0 - 1)	0 (0 - 2)	0.6337
AIS Chest	3 (0 -3.5)	3.5 (2.5 -4.5)	0.1266
AIS Abdomen	0 (0 - 2)	0 (0 - 0.5)	0.1338
AIS Extremeties/Pelvis	2.5 (0 - 3)	2 (2 - 3)	0.7421
AIS External	0 (0 - 0)	0 (0 - 0.5)	> 0.9999
CK	300 (153 - 651)	248 (195 - 672)	0.8171
LDH	355 (242 - 567)	340 (303 - 411)	0.5551
Troponin T	12 (9 - 24)	15 (7 - 54)	0.8248
Base Excess	-1.2 (-3.3 - 0.9)	-1.3 (-3.2 - -0.2)	0.7478
Lactate	2 (1 - 3)	2 (1 - 4)	0.6693
GCS	12 (5 - 15)	11 (5 - 14)	0.9124
Heartrate	85 (80 - 102)	65 (54 - 70)	**0.0050**
Systolic BP	120 (100 - 140)	129 (95 - 143)	0.8892
Diastolic BP	75 (60 - 80)	81 (55 - 90)	0.9184

Supplementary Table 2: Patient cohort surviving until discharge versus non-survivers. Descriptive measures and available laboratory values at admission of the group that survived to hospital discharge and the group that did not. (unless otherwise specified values represent the median with interquartile range shown in brackets; p values between categorical variables assessed using fisher´s exact test; p values between continuous variables assessed with One-way ANOVA; TBI= traumatic brain injury; AIS = abbreviated injury scale; ISS = Injury Severity Score; NISS = New Injury Severity

Score; CK = creatine kinase activity in U/l; LDH = Lactate Dehydrogenase activity in U/l; Lactate in mmol/l; Troponin T in ng/l ;GCS= Glasgow Coma Scale; BP = Blood Pressure in mmHg)

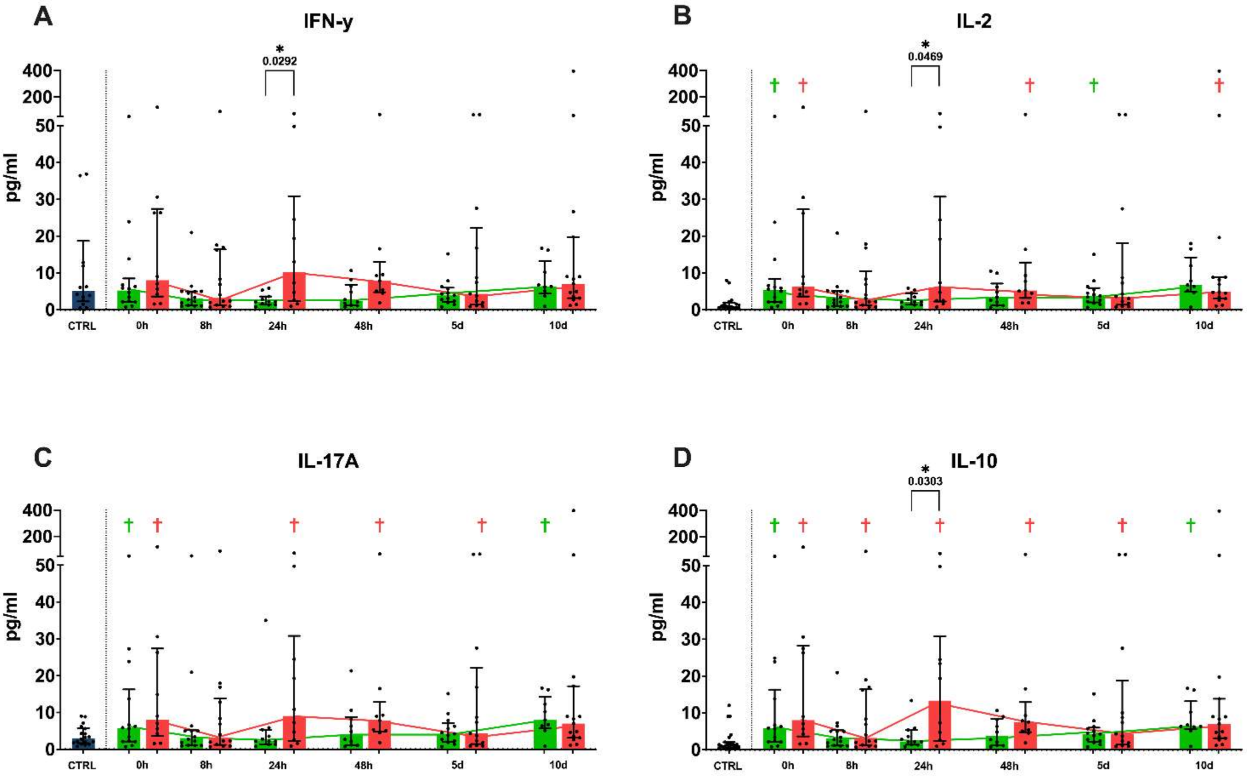

Supplementary Figure 2: Cytokine concentrations in serum for multi organ dysfunction syndrome (MODS) outcome. Systemic IFN-γ (A), IL-2 (B), IL-17 (C) and IL-10 (D) concentrations shown in pg/ml. (Blue = healthy control population, green = did not develop MODS, red= developed MODS during hospital stay; * = $p < 0.05$ between the two outcome groups at the timepoint; † = $p < 0.05$ compared to healthy control population, Mann-Whitney-U-Test; median ± interquartile range)

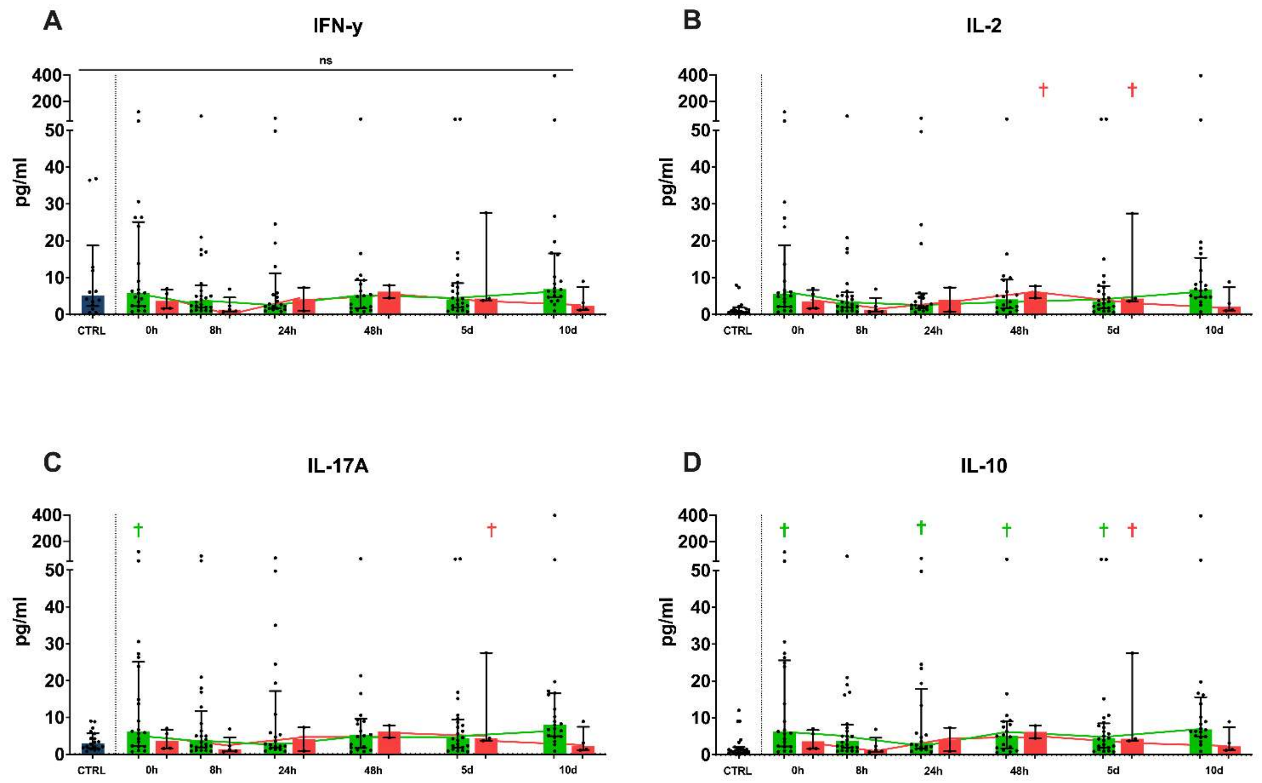

Supplementary Figure 2: Cytokine concentrations in serum for survival outcome. Systemic IFN-γ (A), IL-2 (B), IL-17 (C) and IL-10 (D) concentrations shown in pg/ml. (Blue = healthy control population, green = survived to discharge, red= died in hospital; † = $p < 0.05$ compared to healthy control population, ns = no significant differences between any timepoints or compared to healthy controls; Mann-Whitney-U-Test; median ± interquartile range)

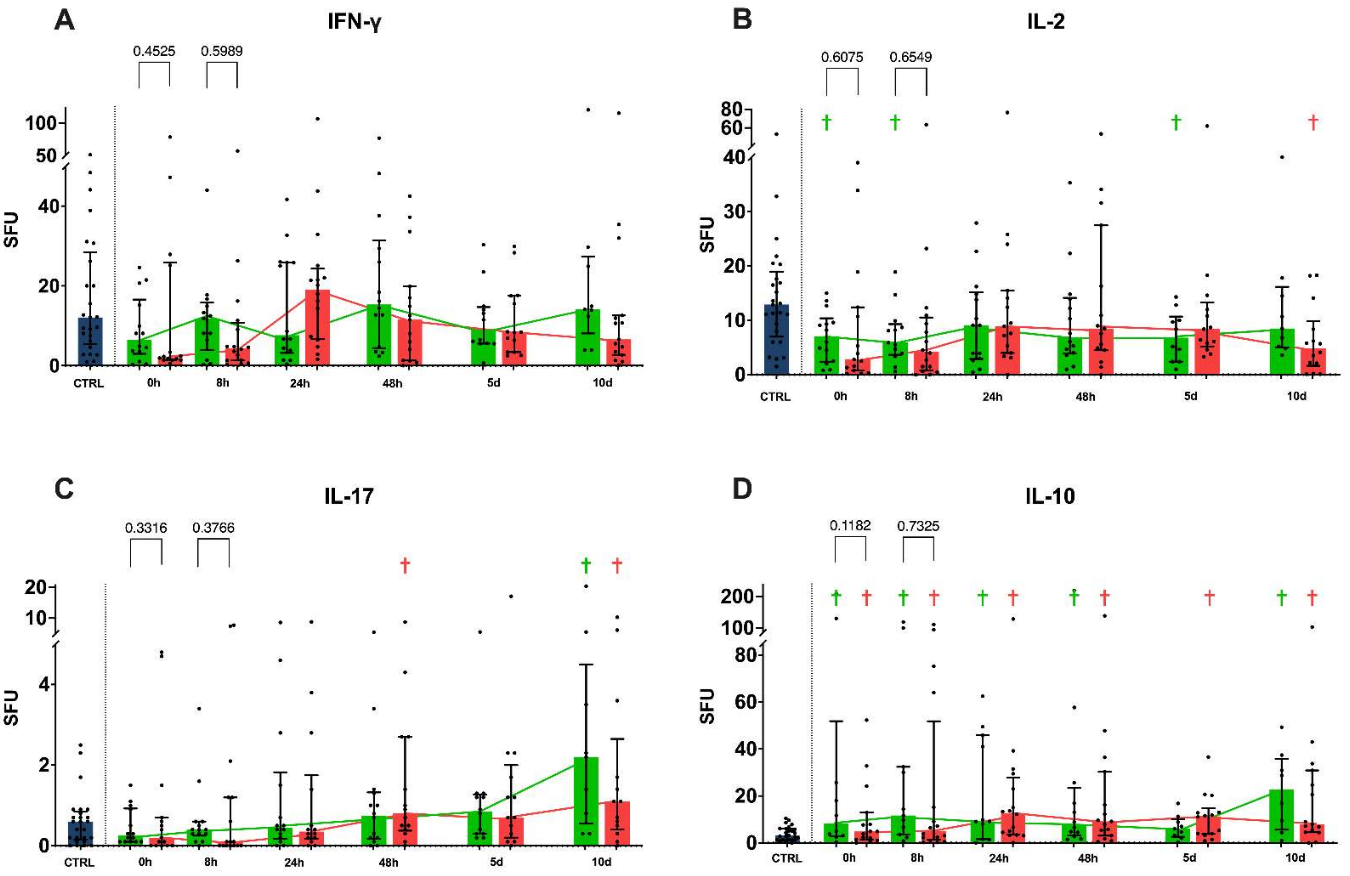

Supplementary Figure 3: Functional cytokine release in ELISpot depending on the development of MODS during hospital stay. Anti CD3/28 stimulated ELISpot spot forming units (SFU) per 1µl of whole blood for IFN-γ (A), IL-2 (B), IL-17 (C) and IL-10 (D) shown in absolute numbers. p values between the two outcome groups shown for all 0 and 8h timepoints. (Blue = healthy control population, green = did not develop MODS, red= developed MODS during hospital stay; † = $p < 0.05$ compared to healthy control population; Mann-Whitney-U-Test; median ± interquartile range)

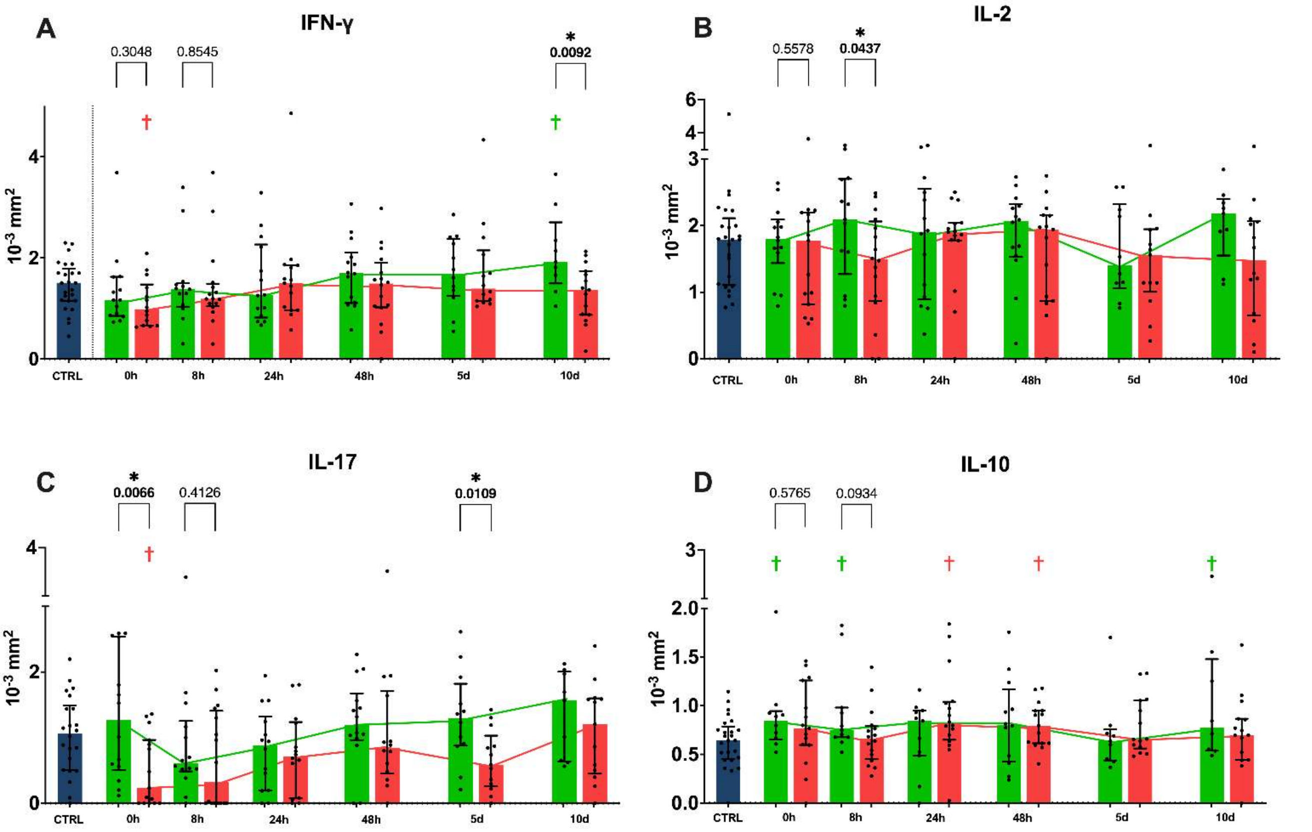

Supplementary Figure 4: Functional cytokine release in ELISpot depending on the development of MODS during hospital stay. Anti CD3/28 stimulated ELISpot spot size for IFN-γ (A), IL-2 (B), IL-17 (C) and IL-10 (D) shown in 10^{-3} mm^2 . p values between the two outcome groups shown for all 0 and 8h timepoints. (Blue = healthy control population, g green = did not develop MODS, red= developed MODS during hospital stay; † = $p < 0.05$ compared to healthy control population; * = $p < 0.05$ between the two outcome groups at the timepoint; Mann-Whitney-U-Test; median ± interquartile range)

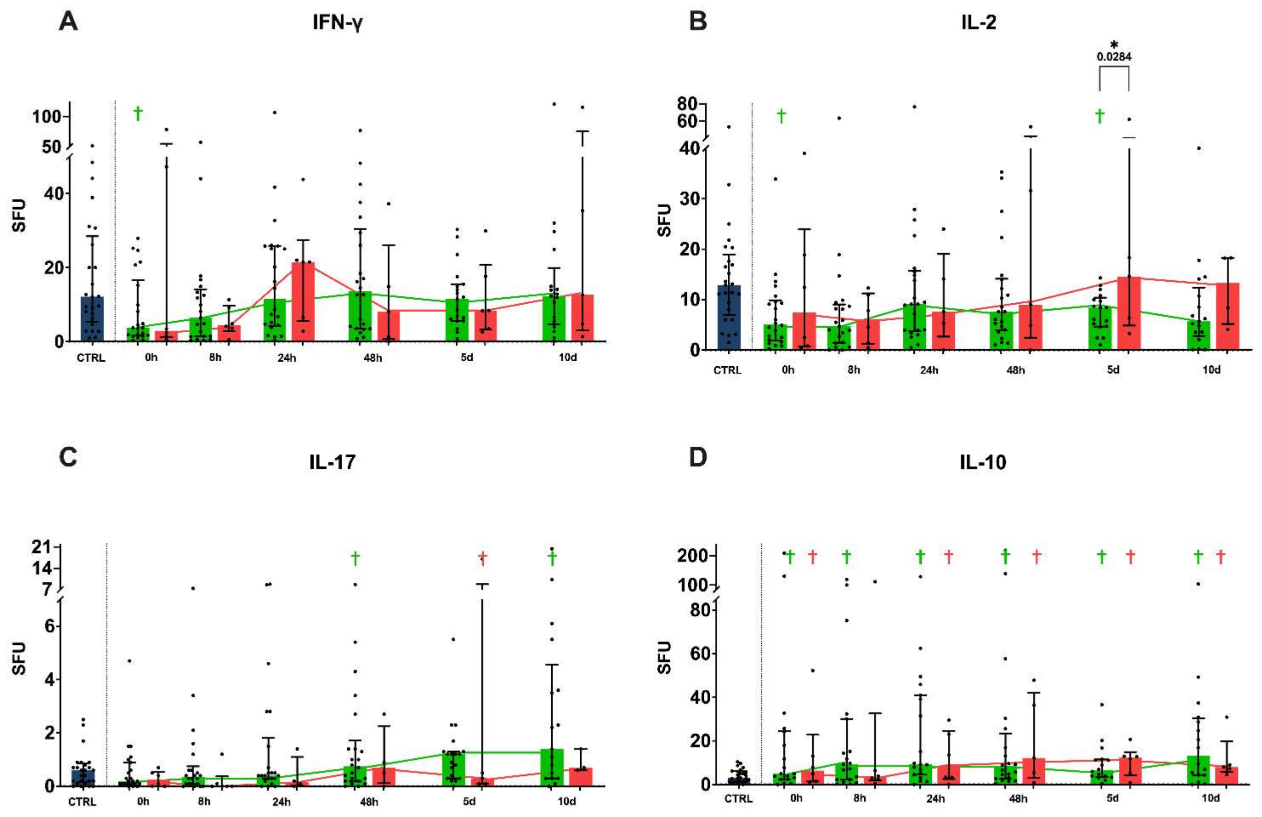

Supplementary Figure 5: Functional cytokine release in ELISpot depending on in-hospital survival. Anti CD3/28 stimulated ELISpot spot forming units (SFU) per 1 µl of whole blood for IFN-γ (A), IL-2 (B), IL-17 (C) and IL-10 (D) shown in absolute numbers. (Blue = healthy control population, green = survived to discharge, red= died in hospital; † = p < 0.05 compared to healthy control population; * = p < 0.05 between the two outcome groups at the timepoint; Mann-Whitney-U-Test; median ± interquartile range)

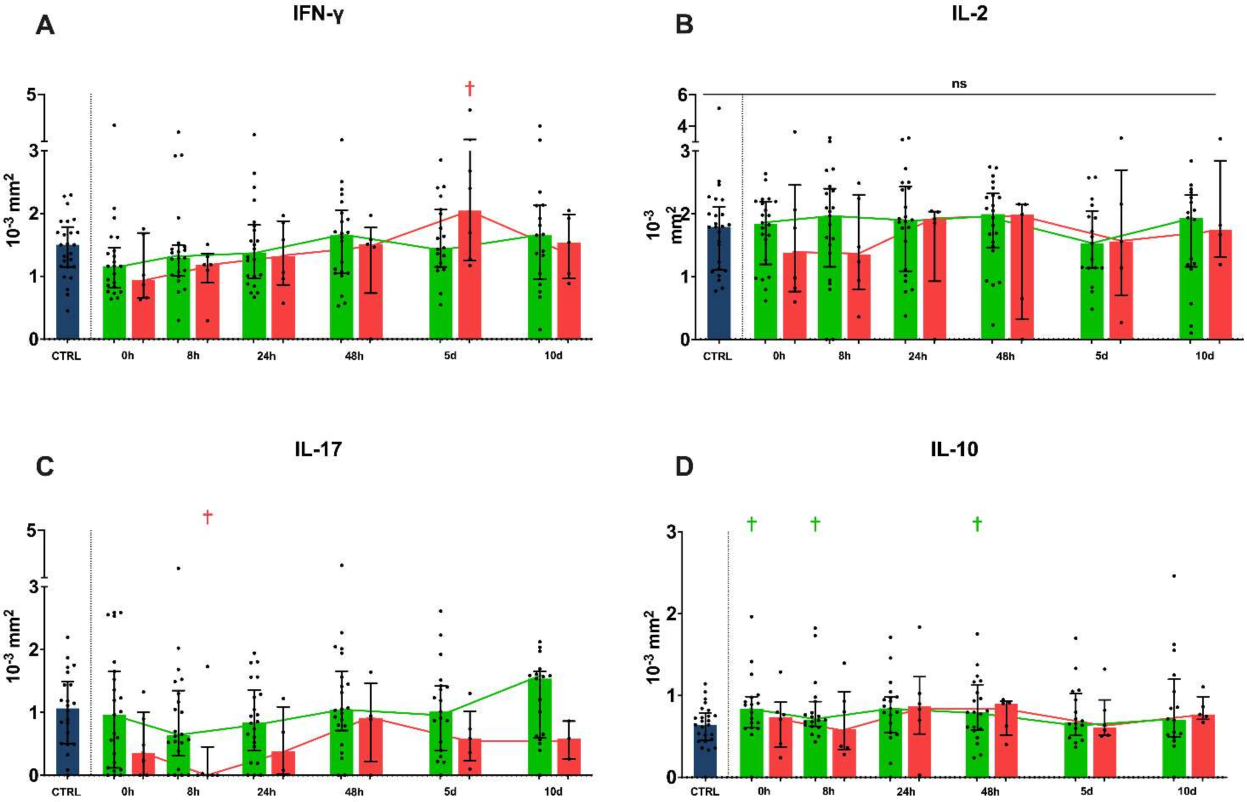

Supplementary Figure 6: Functional cytokine release in ELISpot depending on in-hospital survival. Anti CD3/28 stimulated ELISpot spot size for IFN-γ (A), IL-2 (B), IL-17 (C) and IL-10 (D) shown in 10^{-3} mm^2. (Blue = healthy control population, green = survived to discharge, red= died in hospital; † = $p < 0.05$ compared to healthy control population; ns = no significant differences between any timepoints or compared to healthy controls; Mann-Whitney-U-Test; median ± interquartile range)

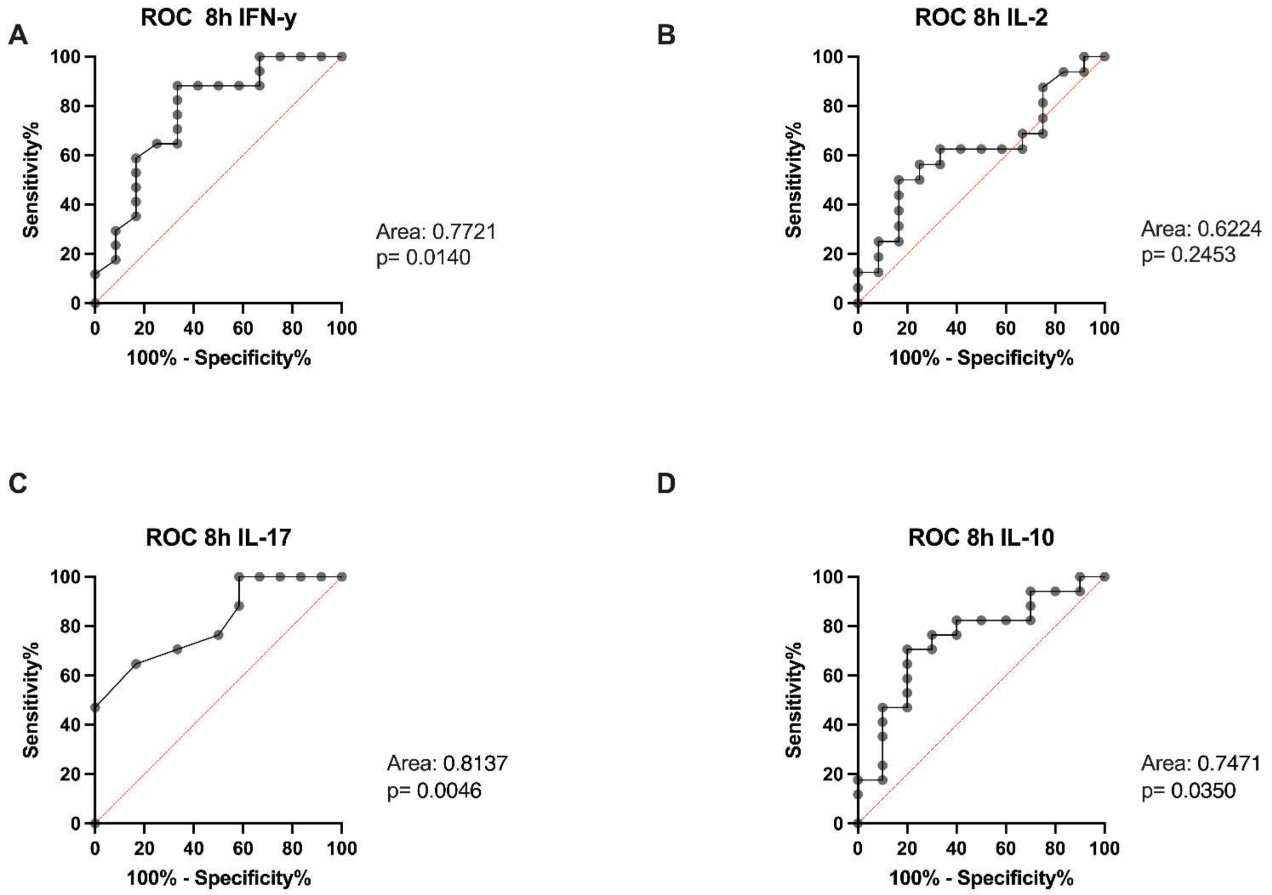

Supplemental Figure 7: Receiver-Operating-Characteristics (ROC) for ELISpot spot forming units at 8 hours for IFN-γ (A), IL-2 (B), IL-17 (C) and IL-10 (D). (Area under curve (AUROC) shown in the graph, threshold for significance $p \leq 0.05$)

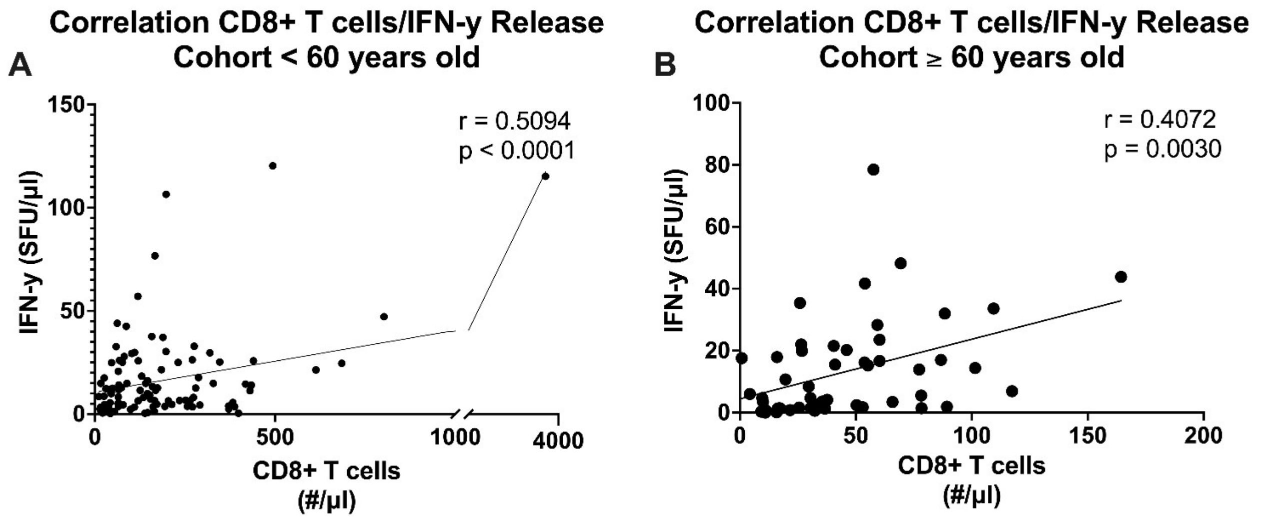

Supplemental Figure 8: Correlation between blood CD8+ T cell numbers and functional anti CD3/28 stimulated IFN-γ release depending on age. Result of Pearson´s correlation test between whole blood CD8 T cells per µl and IFN-γ spot forming untis (SFU) for patients under 60 years of age (A) and aged 60 and above (B). (Threshold for significance $p < 0.05$)

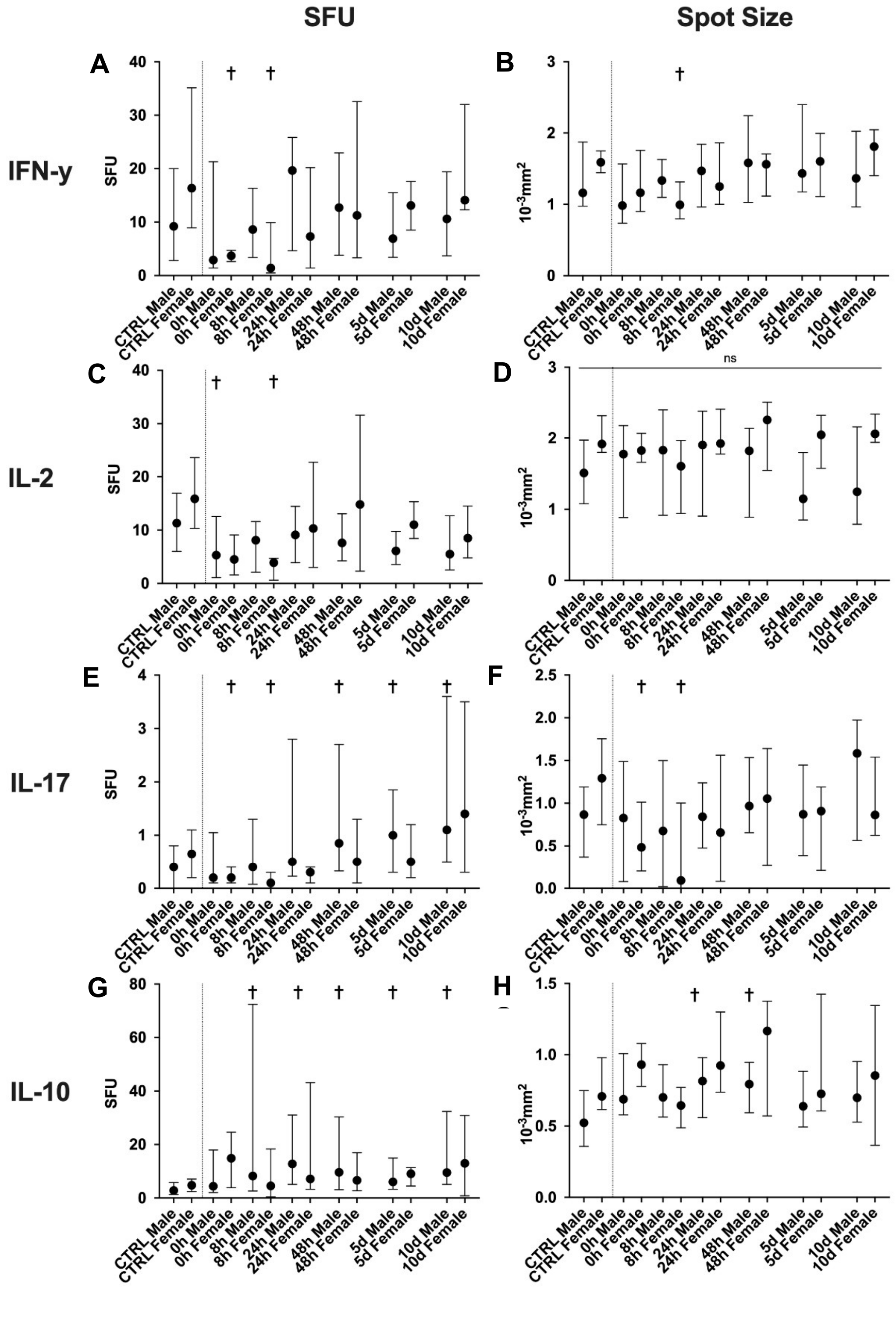

Supplemental Figure 9: Overall dynamic of T cell-specific CD3/28-stimulated cytokine release in ELISpot assay grouped by patient gender. Counted spot forming units (SFU) per 1µl of whole blood of IFN-γ (A), IL-2 (C), IL-17 (E), and IL-10 (G) in absolute numbers. Mean of the size of all counted spots (Spot Size) for IFN-γ (B), IL-2 (D), IL-17 (F) and IL-10 (H) shown in 10^{-3} square millimeters. (median ± interquartile range; ns = no statistically significant differences between the genders at the

specific timepoint, † = $p < 0.05$ compared to healthy control population Mann-Whitney-U-Test, threshold for significance $p < 0.05$).
